# The Synergistic Effects of rhArg with Bcl-2 Inhibitors or Metformin Co-Treatment in Multiple Cancer Cell Models

**DOI:** 10.3390/cells15020164

**Published:** 2026-01-16

**Authors:** Lai-Pan Sze, Vicky Mei-Ki Ho, Wing-Ki Fung, Kin-Ho Law, Yifan Tu, Yik-Hing So, Sai-Fung Chung, Wing-Leung Wong, Zhen Liu, Alisa Sau-Wun Shum, Leo Man-Yuen Lee, Yun-Chung Leung

**Affiliations:** 1 Department of Applied Biology and Chemical Technology, The Hong Kong Polytechnic University, Hung Hom, Kowloon, Hong Kong, Chinawing.leung.wong@polyu.edu.hk (W.-L.W.); 2 Lo Ka Chung Research Centre for Natural Anti-Cancer Drug Development, The Hong Kong Polytechnic University, Hung Hom, Kowloon, Hong Kong, China; 3 State Key Laboratory of Marine Food Processing and Safety Control, College of Food Science and Engineering, Ocean University of China, Qingdao 266404, China; 4 School of Biomedical Sciences, Faculty of Medicine, The Chinese University of Hong Kong, Hong Kong, China

**Keywords:** recombinant human arginase (rhArg), Bcl-2 inhibitor, ABT263, drug combination, anticancer, metformin

## Abstract

Background: Recombinant human arginase (rhArg) has been proven to exhibit an anticancer effect via arginine starvation. To further improve the efficacy of rhArg, we examined the feasibility of a combination strategy with Bcl-2 inhibitors (ABT263 and ABT199) or an antidiabetic drug (metformin) and investigated the mechanistic basis for these strategies. Methods: The combination effects were evaluated in a panel of human cancer cell lines modeling pancreatic ductal carcinoma (PDAC), triple-negative breast cancer (TNBC), colorectal cancer (CRC) and glioblastoma (GBM). Western blot analysis was used to evaluate the expression of apoptotic and cell cycle markers. MTT assay was used to evaluate the combination efficacy. Flow cytometric assays were used to investigate the apoptotic and cell cycle effects. Results: The combination of rhArg with sublethal doses of ABT263 significantly induced dose-dependent apoptosis, with elevated expression of apoptotic markers and a CI of 0.47 in U251. The combination inhibited CDK2 and cyclin A expression, indicating that the observed synergy also resulted from cell cycle arrest. We also found that rhArg + metformin was synergistic in a time-dependent manner. Compared to other amino acid depletion agents, rhArg + ABT263 was the most favorable combination pair. Conclusions: The combination of rhArg and ABT263 enhanced apoptosis and cell cycle arrest, demonstrating a potential broad-spectrum antitumor strategy.

## 1. Introduction

Pancreatic ductal carcinoma (PDAC) is asymptomatic in its early stage, leading to a late diagnosis when a curative approach is rarely feasible. Consequently, PDAC has a high mortality rate and a five-year survival rate below 10% [1,2]. Triple-negative breast cancer (TNBC) is a subtype of breast cancer that does not express estrogen (ER), progesterone (PR) and human epidermal growth factor (HER2+) receptors [3]. The lack of hormonal receptors renders targeted therapies ineffective in TNBC. Its aggressive nature accounts for over 50% of breast cancer mortality [4]. Patients with late-stage colorectal cancer (CRC), the third most common cancer and second leading cause of cancer mortality globally, are primarily managed with postoperative chemotherapy [5]. Glioblastoma accounts for more than 60% of diagnosed brain tumors, with an overall survival of around 15 months [6]. Therapeutic effects in glioblastoma are critically limited by the blood–brain barrier (BBB). Therefore, these malignancies present distinct therapeutic hurdles. Although chemotherapy is a widely accepted approach in treating these cancers, only a portion of patients respond to it. For example, more than 50% of TNBC patients are resistant to chemotherapy [7]. Patients who initially respond to the treatment will eventually develop local recurrence or metastatic disease. Therefore, there is a pressing need to develop alternative therapeutic strategies that encompass the majority of cancers.

Arginine depletion therapy is an emerging therapeutic approach for cancer treatment. A lack of expression of the critical urea cycle enzymes arginosuccinate synthetase (ASS) or ornithine transcarboxylase (OTC) renders cancer cells auxotrophic to arginine. There are multiple arginine depletion agents available and our group has previously reported and developed a recombinant human arginase (rhArg) which is applicable in multiple types of cancers [8]. Arginine deiminase (ADI) converts arginine to citrulline, making it ineffective against ASS-positive cancer cells that can regenerate arginine [9]. In contrast, the ornithine produced by rhArg requires both OTC and ASS for arginine regeneration. This dual dependency makes rhArg effective against ASS-positive cells [8]. Under a paucity of arginine, cancer cells experience cell cycle arrest, autophagy and subsequent apoptosis [10,11]. In spite of this, a phase III clinical trial confirmed that Pegargiminase (ADI-PEG20) monotherapy failed to improve overall survival in advanced hepatocellular carcinoma (NCT01287585). Investigators attributed this lack of efficacy to neutralizing antibodies induced by Pegargiminase, revealing a fundamental challenge of an arginine depletion strategy [12].

Combination therapy is of particular interest, as it combines drugs with diverse mechanisms to redirect current drugs to new indications and reduce their dosages [13]. In a phase III clinical trial, a regimen combining Pegargiminase (ADI-PEG20) with pemetrexed and platinum drugs successfully increased overall survival by 2 months in patients with malignant pleural mesothelioma (NCT02709512) [14]. Therefore, we sought to combine rhArg with several drugs that are either already marketed or under development. ABT263 is a dual Bcl-2 inhibitor that targets two anti-apoptotic proteins, Bcl-2 and Bcl-xL. It is an ongoing phase II investigational drug, and multiple clinical trials have been initiated to evaluate its efficacy against various cancer types. The commercially available drugs used in this study were the Bcl-2 inhibitor ABT199 (Venetoclax) and the antidiabetic agent metformin. The frequent overexpression of anti-apoptotic proteins in cancers highlights their oncogenic function and has driven the development of Bcl-2-targeted therapies [15]. Activation of adenosine monophosphate-activated protein kinase (AMPK) by metformin was believed to be one of metformin’s anticancer mechanisms [16]. To date, the combination of rhArg with Bcl-2 inhibitors or metformin has not been systematically investigated for improving outcomes.

This study aimed to investigate the combination of rhArg with Bcl-2 inhibitors or metformin in four cancer cell lines: Panc-1/Gemcitabine resistance (Panc-1R) (PDAC), MDA-MB-231 (TNBC), HT-29 (CRC) and U251 (glioblastoma). We aimed to find out whether combining rhArg with other chemical entities would result in a general cytotoxic effect in multiple cancer cell models. The potential synergistic anticancer effect of the combination and the biological outcomes were investigated.

## 2. Materials and Methods

### 2.1. Recombinant Human Arginase (rhArg), Arginine Deiminase (ADI) and Asparaginase Preparation

The arginase used in this study was recombinant human arginase (rhArg) [17]. The use of ADI and asparaginase have been reported previously [18,19].

### 2.2. Cell Culture

Colorectal cell line HT-29 and triple-negative breast cancer cell line MDA-MB-231 were purchased from the American Type Culture Collection (ATCC) (Manassas, VA, USA). The pancreatic ductal carcinoma Panc-1/gemcitabine resistance (Panc-1R) cell line was a gift from Prof. Wong Wing Leung from The Hong Kong Polytechnic University and was purchased from Xiamen Immocell Biotechnology Co., Ltd. (Cat. IMD-015, Xiamen, China). The glioblastoma U251 cell line was a gift from Prof. Giberto Leung from The University of Hong Kong. The cell lines were cultured in Dulbecco’s Modified Eagle Medium (DMEM), high glucose, GlutaMAX™ (HT-29 and Panc-1R) supplemented with 10% fetal bovine serum (Gibco, Waltham, MA, USA), 100 units/mL streptomycin sulfate (Gibco), 100 units/mL penicillin G sulfate (Gibco) and 1 mM sodium pyruvate (Gibco) at 37 °C and 5% CO_2_; Dulbecco’s Modified Eagle Medium (DMEM), high glucose, GlutaMAX™ (MDA-MB-231) supplemented with 10% fetal bovine serum (Gibco), 100 units/mL streptomycin sulfate (Gibco) and 100 units/mL penicillin G sulfate (Gibco) at 37 °C and 5% CO_2_; and Roswell Park Memorial Institute (RPMI) 1640 Medium (U251) supplemented with 10% fetal bovine serum (Gibco), 100 units/mL streptomycin sulfate (Gibco) and 100 units/mL penicillin G sulfate (Gibco) at 37 °C and 5% CO_2_.

### 2.3. Chemicals and Reagents

GCN2iB (Cat. HY-112654), ABT199 (Cat. HY-15531) and ABT263 (Cat. HY-10087) were purchased from MedChemExpress (Monmouth Junction, NJ, USA). Metformin hydrochloride (Cat. 13118) was purchased from Cayman Chemical (Ann Arbor, MI, USA).

### 2.4. Cell Viability and Analysis of Drug Combination

Cell viability was evaluated by 3-[4,5-dimethylthiazol-2-yl]-2,5 diphenyl tetrazolium bromide (MTT) (Cat. M6494, Invitrogen, Thermo Fisher, Carlsbad, CA, USA) assay as described previously [8]. A total of 100 μL of the culture medium that contained 1.5 × 10^3^ cells was seeded in a 96-well plate. After overnight incubation, the cells were treated with drugs in serial dilution at a 1:1 ratio for 72 h. MTT was added with a final concentration of 0.5 mg/mL per well for 4 h. After 4 h, the medium was then removed and 100 µL of DMSO was dispensed to each well to solubilize the purple dye, and absorbance was measured at 570 nm. Drug interactions between rhArg and various chemical entities were analyzed using the median-effect method. Compounds were combined at a fixed ratio based on their IC_50_ values [20], and synergy was quantified by the combination Index (CI), where CI < 1, =1 and >1 indicate synergy, additivity and antagonism, respectively.

### 2.5. Western Blot Analysis

After drug treatment, cells were harvested in RIPA buffer (Cat. R0278, Sigma Aldrich, St. Louis, MO, USA) supplemented with protease (Cat. 78430, Thermo Fisher, Rockford, IL, USA) and phosphatase (Cat. 78428, Thermo Fisher, Rockford, IL, USA) inhibitors. Protein lysates were subjected to sodium dodecyl sulfate–polyacrylamide gel electrophoresis and transferred to Immobilon^®^-P PVDF Membrane (PVDF) (Cat. IPVH00010, Merck Millipore, Darmstadt, Germany) for Western blot analysis using the following primary antibodies: BAX (cst-2772), GAPDH (cst-2118), cleaved caspase 3 (cst-9664) and PARP (cst-9542) from Cell Signaling Technology (Danvers, MA, USA) or cyclin A (sc-239), CDK2 (sc-6248) and Mcl-1 (sc-12756) from Santa Cruz Biotechnology (Paso Robles, CA, USA). Specific protein bands were visualized with Immobilon Western Chemiluminescent HRP Substrate (Cat. WBKLS0500, Merck Millipore, Darmstadt, Germany) and the Chemi Doc Imaging System (Bio-Rad, Hercules, CA, USA).

### 2.6. Cell Cycle Analysis

Following trypsinization, the treated cells were harvested, washed with ice cold PBS and fixed in ice-cold 70% ethanol. The fixed samples were stored at −20 °C for later analysis. Before analysis, ethanol was discarded and the cells were washed twice with ice-cold PBS. The fixed cells were incubated with BD Pharmingen™ PI/RNase Staining Buffer (Cat. 550825, BD Biosciences, San Jose, CA, USA) according to the manufacturer’s instructions and subjected to flow cytometric analysis using BD FACSVia (BD Biosciences, Franklin Lakes, NJ, USA). For every sample, 10,000 events were collected and subsequently analyzed with ModFit software (Version 5.0, Verity Software House, Topsham, ME, USA).

### 2.7. Annexin V Apoptosis Assay

Cells were seeded in 6-well plates (Thermo Fisher, Carlsbad, CA, USA) and exposed to a range of drug concentrations, both as single agents and in combination. Following treatment, all cells, including those in suspension and those remaining adherent, were harvested via trypsinization. Apoptosis was quantified with the FITC Annexin V Apoptosis Detection Kit I (BD Pharmingen™, Cat. 556547, Franklin Lakes, NJ, USA). For every sample, 10,000 events were recorded on a BD FACSVia flow cytometer, and the resulting data were processed using the BD FACSVia software (Version 1.1, BD Biosciences, Franklin Lakes, NJ, USA).

### 2.8. Statistical Analysis

All experimental data represent the mean of at least three independent replicates. Data analysis was performed using GraphPad Prism 9.4.1 (GraphPad Software, San Diego, CA, USA), with statistical significance and analysis indicated in the corresponding figure or table legends.

## 3. Results

### 3.1. Antiproliferative Effects of rhArg, Bcl-2 Inhibitors and Metformin in the Cancer Cell Lines

Our previous studies have reported the broad anticancer spectrum of rhArg. As shown in Table 1, all in vitro models were sensitive to rhArg treatment. Although the IC_50_ value of rhArg in U251 was around 3- to 4-fold higher than other models, rhArg significantly suppressed cancer cell proliferation at sub-microgram levels. To compare the efficacy of rhArg with other amino acid depletion agents, we further included ADI [19]. The results in Table 1 indicated that U251 was resistant to ADI. Although we did observe a certain degree of growth inhibition at the maximum given dose of ADI (10 µg/mL) in U251, the percentages of viable cells at this dose were larger than 50% and, therefore, termed “resistant”. The dual Bcl-2 inhibitor ABT263 was slightly more potent than its single Bcl-2 inhibitor counterpart, ABT199, suggesting that targeting multiple anti-apoptotic proteins had a better antiproliferation effect [21]. We also included the GCN2 inhibitor GCN2iB, as previous studies have reported a synergistic effect between GCN2 inhibition and arginine depletion in hepatocellular carcinoma (HCC) [22]. IC_50_ values of GCN2iB and metformin agreed with the reported literature [23,24].

### 3.2. Combination of rhArg with ABT263 Exhibited Pronounced Synergistic Activity

The synergistic potential of rhArg in combination with either Bcl-2 inhibitors or metformin was assessed across multiple in vitro cancer models using median-effect analysis at equipotent drug concentrations. As detailed in Table 2, the most robust synergy was identified in U251 cells treated with the rhArg and ABT263 combination. This pair demonstrated a combination index (CI) of 0.47, denoting strong synergism [25]. Furthermore, the dose reduction index (DRI) for both agents exceeded 4, implying that each drug’s effective dose could be lowered over 4-fold while maintaining anticancer efficacy. Notably, the rhArg + ABT263 combination also yielded CI values below 1 and DRI values above 2 in the three other cell lines tested, with Panc-1R exhibiting the lowest sensitivity to this treatment (CI = 0.90). The combination of rhArg with ABT199 was also synergistic; HT-29 was the most sensitive cell line with a CI of 0.69 and U251 was the least sensitive to this combination (CI = 0.82). These two combinations were synergistic in four in vitro models, signifying a favorable combination between rhArg and Bcl-2 inhibitors.

The combination of rhArg with metformin exhibited a time-dependent synergistic effect. This combination was antagonistic (>1) in HT-29 and MDA-MB-231 at 48 h but became synergistic after 96 h incubation. The combination effect, however, was less pronounced compared to that of ABT263.

### 3.3. rhArg + ABT263 Exhibited Significant Cytotoxic Effect While rhArg + Metformin Blunted Colony Formation Ability in HT-29

**Figure 1:**
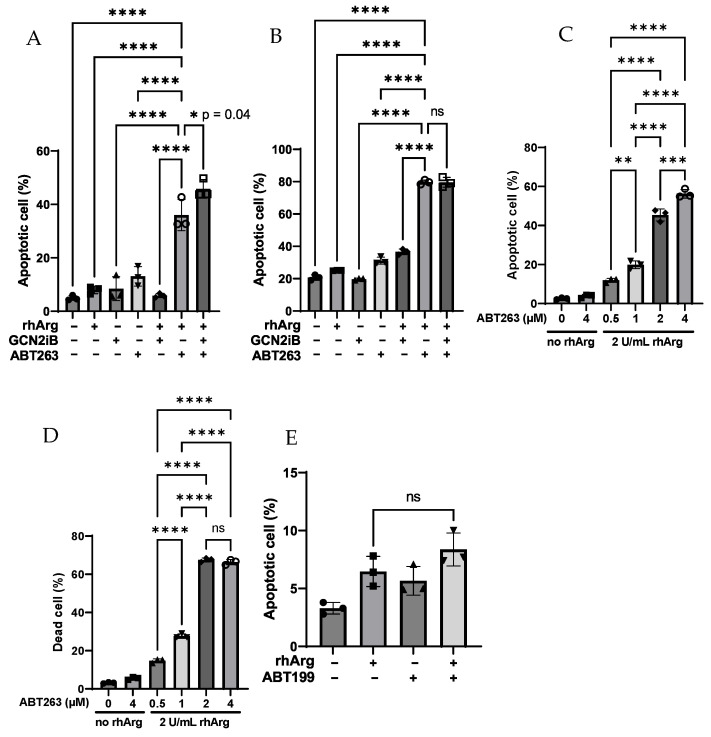
Annexin V/PI assessment of apoptosis in HT-29 cells. Summary of apoptosis assay data of HT-29 cells cultured in control, rhArg, 2 μM GCN2iB, 2 μM ABT-263 or their combinations for (**A**) 3 days and (**B**) 7 days. Cells were treated with escalating doses of ABT263 in combination with a fixed dose of rhArg (2 U/mL) for 3 days and (**C**) the percentage of apoptotic cells and (**D**) the percentage of total dead cells were quantified. (**E**) Cells were treated by either 2 U/mL rhArg and 4 µM ABT199 alone or in combination, respectively, for 3 days and the percentage of apoptotic cells was quantified. Apoptotic cells = early apoptotic cells + late apoptotic cells. Dead cells = necrotic cells + apoptotic cells. Statistical analysis was carried out by one-way ANOVA with a post hoc Tukey test (*n* = 3). (ns) denotes non-significant, (*) denotes *p* < 0.05, (**) denotes *p* < 0.01, (***) denotes *p* < 0.001 and (****) denotes *p* < 0.0001. The raw representative dot plot is shown in Supplementary Figure S2.

In light of our findings and supported by the literature demonstrating that ABT263 eradicated cancer cells cultured under prolonged amino acid paucity, we assessed whether the synergistic effect of rhArg with Bcl-2 inhibitors was due to apoptosis [22]. We used 2 U/mL (10 µg/mL) of rhArg in the following experiments to reduce extracellular arginine to a low level (<10 µM) within 30 min (Supplementary Figure S1). As arginine depletion by rhArg is a time- and dose-dependent enzymatic reaction (Supplementary Figure S1), we aimed to rule out these variables in this study. We first combined three drugs, rhArg + ABT263 + GCN2iB, and assessed the efficacy in HT-29 cells in a time-dependent manner. Surprisingly, as depicted in Figure 1A,B, we observed that rhArg + ABT263 was enough to induce a comparable percentage of apoptotic cells compared to that of the three-drug combination. The percentage of apoptotic cells was increased in a time-dependent manner from around 40% at day 3 to around 80% at day 7. Although the three-drug combination offered little improvement compared to rhArg + ABT263 at day 3 (rhArg + ABT263: 36%, three drugs: 46%, *p* = 0.04) (Figure 1A), its effect became comparable at day 7 (Figure 1B).

**Figure 2:**
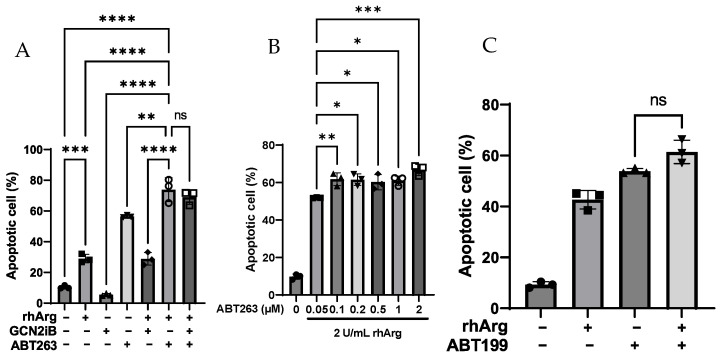
Annexin V/PI assessment of apoptosis in MDA-MB-231 cells. (**A**) Summary of apoptosis assay data of MDA-MB-231 cells cultured in control, rhArg, 2 μM GCN2iB, 2 μM ABT-263 or their combinations for 3 days. (**B**) Cells were treated with escalating doses of ABT263 in combination with a fixed dose of rhArg (2 U/mL) for 3 days and the percentage of apoptotic cells was quantified. (**C**) Cells were treated by either 2 U/mL rhArg alone, 4 µM ABT199 alone or their combination, respectively, for 3 days and the percentage of apoptotic cells was quantified. Statistical analysis was carried out by one-way ANOVA with a post hoc Tukey test (*n* = 3). (ns) denotes non-significant, (*) denotes *p* < 0.05, (**) denotes *p* < 0.01, (***) denotes *p* < 0.001 and (****) denotes *p* < 0.0001. The raw representative dot plot is shown in Supplementary Figure S3.

**Figure 3:**
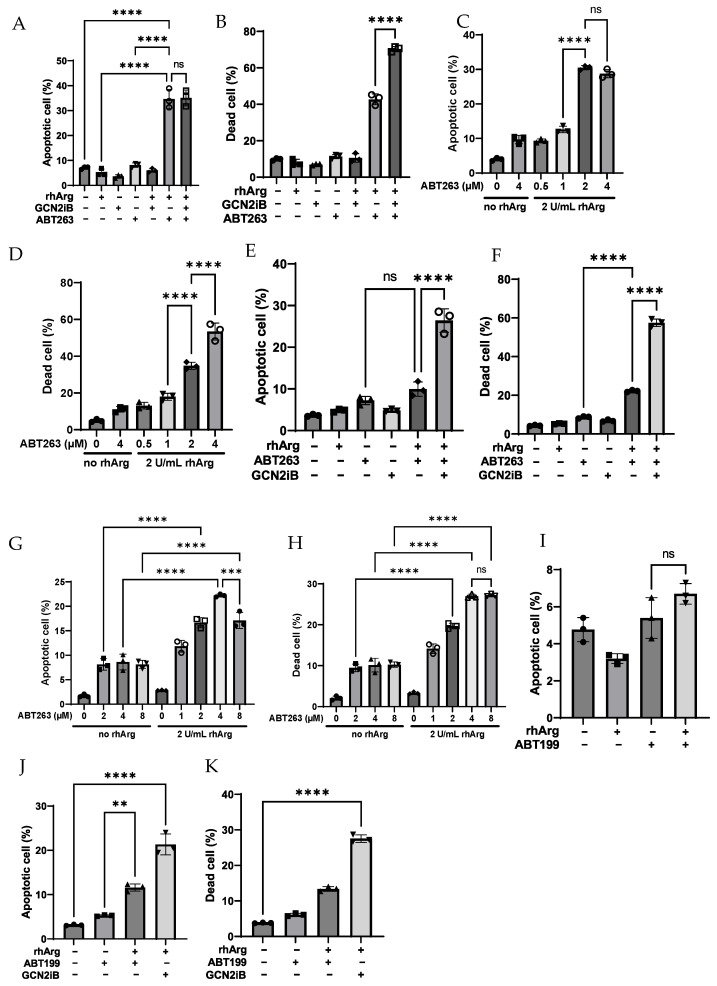
Annexin V/PI assessment of apoptosis in U251 and Panc-1R cells. (**A**) Apoptosis and (**B**) total dead cells in U251 after 3-day treatment with control, rhArg, 2 μM GCN2iB, 2 μM ABT-263 or their combinations. U251 cells were treated with escalating doses of ABT263 in combination with a fixed dose of rhArg (2 U/mL) for 3 days and (**C**) the percentage of apoptotic cells and (**D**) the percentage of total dead cells were quantified. (**E**) Apoptosis and (**F**) total dead cells in Panc-1R after 3-day treatment with control, rhArg, 4 μM GCN2iB, 4 μM ABT-263 or their combinations. Panc-1R cells were treated with escalating doses of ABT263 in combination with a fixed dose of rhArg (2 U/mL) for 3 days and (**G**) the percentage of apoptotic cells and (**H**) the percentage of total dead cells were quantified. (**I**) U251 cells and (**J**,**K**) Panc-1R cells were treated with either 2 U/mL rhArg alone, 10 µM ABT199 alone, 4 µM GCN2iB (Panc-1R only) or their combination, respectively, for 3 days and the percentage of apoptotic cells and dead cells were quantified. Apoptotic cells = early apoptotic cells + late apoptotic cells. Dead cells = necrotic cells + apoptotic cells. Statistical analysis was carried out by one-way ANOVA with a post hoc Tukey test (*n* = 3). (ns) denotes non-significant, (**) denotes *p* < 0.01, (***) denotes *p* < 0.001 and (****) denotes *p* < 0.0001. The raw representative dot plot is shown in Supplementary Figure S4.

To determine whether the combination of rhArg + ABT263 induced a similar effect in other in vitro models, we treated these cell lines with the same strategy. As depicted in Figure 2A (MDA-MB-231), Figure 3A,B (U251) and Figure 3E,F (Panc-1R), rhArg + ABT263 also induced significant apoptosis in these three cell lines. For the results of U251 and Panc-1R, we found that the combination not only increased apoptotic cells but also increased necrotic cells. This was not surprising as it was reported that apoptotic cells could become necrotic due to “secondary necrosis” [26]. Therefore, during quantification of cell death, we calculated “dead cells” by summing necrotic cells with apoptotic cells. In MDA-MB-231, rhArg + ABT263 induced more than 80% apoptotic cells compared to that of rhArg alone (29%) and ABT263 alone (56%) (Figure 2A). The percentage of apoptotic cells in U251 was comparable between the three-drug combination and rhArg + ABT263 (Figure 3A), but the percentage of apoptotic cells in Panc-1R treated by the three-drug combination was much higher than that of rhArg + ABT263 (Figure 3E). The percentages of total dead cells in U251 and Panc-1R treated by the three-drug combination were 70% (Figure 3B) and 58% (Figure 3F), respectively, while the dead cell percentages in U251 and Panc-1R treated by rhArg + ABT263 were only 43% (Figure 3B) and 22% (Figure 3F), respectively. Although the three-drug combination outweighed that of rhArg + ABT263 in both U251 and Panc-1R, rhArg + ABT263 was still enough to induce significant cell death. In light of this, we decided to determine whether rhArg + GCN2iB was itself cytotoxic in U251 and Panc-1R. Interestingly, this combination did not induce cell death in U251 (Figure 3B) but had the opposite effect in Panc-1R (Figure 3J,K). rhArg + GCN2iB induced around 28% dead cells (Figure 3K). Therefore, the effect induced by the three-drug combination in Panc-1R (58%) (Figure 3F) was the sum of rhArg + ABT263 (22%) (Figure 3F) and rhArg + GCN2iB (28%) (Figure 3K), while GCN2iB may further potentiate the rhArg + ABT263 effect in U251 (Figure 3B). Furthermore, adding GCN2iB to rhArg + ABT263 offered little improvement in HT-29 and MDA-MB-231, but it may further potentiate the apoptotic effect in U251 and Panc-1R. The unexpected synergy between rhArg and GCN2iB in specific cell lines indicated a cell type-specific response.

We also determined that the cytotoxic effect was dependent on the dose of ABT263. As depicted in Figure 1C,D (HT-29), Figure 2B (MDA-MB-231), Figure 3C,D (U251) and Figure 3G,H (Panc-1R), MDA-MB-231 was very sensitive to the combination, and 0.05 µM ABT263 was still able to elicit the cytotoxic response. The cytotoxic effect was initiated at 2 µM ABT263 in the remaining three cancer cell lines.

In all four cell lines, the combination of a non-cytotoxic concentration of ABT263 (2–4 µM, below IC_50_ values in Table 1) with rhArg greatly potentiated the apoptotic effect. rhArg + ABT263, therefore, exhibited a general cytotoxic effect across all in vitro models.

In light of the results in Table 2, we performed a clonogenic assay in HT-29 to decipher whether the time-dependent synergistic effect of rhArg + metformin was attributed to the antiproliferative effect. As depicted in Supplementary Figure S5, we observed that the countable colony number decreased significantly when HT-29 was treated with rhArg + metformin.

To determine whether the combination of rhArg with the specific Bcl-2 inhibitor ABT199 induced a cytotoxic effect, we combined rhArg with ABT199 at concentrations below its IC_50_ value in the in vitro models. As depicted in Figure 1E (HT-29), Figure 2C (MDA-MB-231), Figure 3I (U251) and Figure 3J (Panc-1R), we did not observe a significant improvement in apoptotic cells when co-administering rhArg with ABT199. The percentages of apoptotic cells following rhArg + ABT199 treatment were comparable to those in the single-agent groups across all four cell lines. The results indicated that dual inhibition of Bcl-2 and Bcl-xL by ABT263 could have sensitized cancer cells to rhArg treatment. As rhArg + ABT199 did not sensitize cancer cells to cell death, the synergism described in Table 2 might be cytostatic in nature.

### 3.4. Cell Cycle Regulatory Effect of rhArg + ABT263

**Figure 4:**
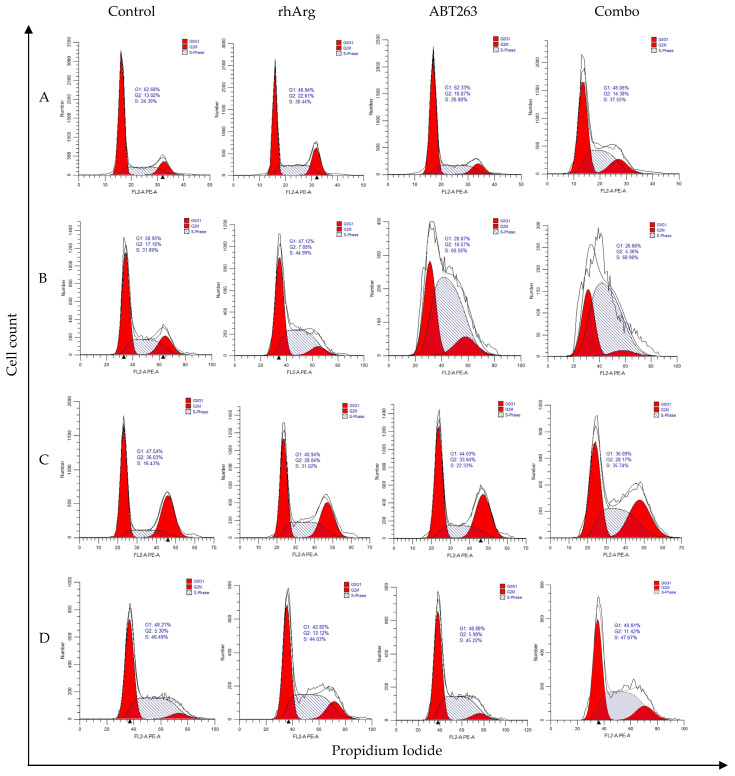

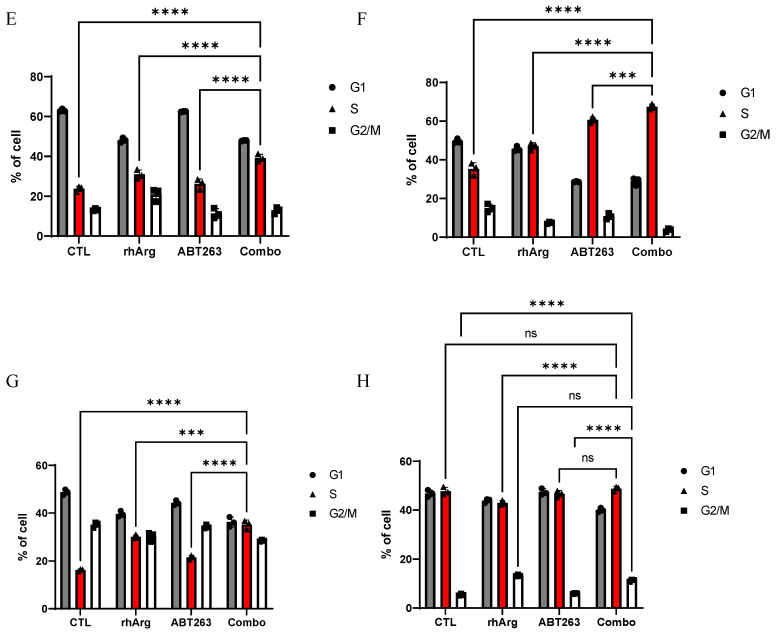
The effects of the 48 h drug combination treatment on cell cycle phase distribution were determined using flow cytometric analysis with propidium iodide (PI) staining and RNase digestion. Condition 1: rhArg: 2 U/mL; ABT263: 2 µM; Combo: 2 U/mL rhArg + 2 µM ABT263. Conditions 2: rhArg: 2 U/mL; ABT263: 4 µM; Combo: 2 U/mL rhArg + 4 µM ABT263. HT-29, MDA-MB-231 and U251 cells were treated with condition 1 for 48 h. Panc-1R cells were treated with condition 2 for 48 h. (**A**,**E**) Cell cycle phase distribution and quantification results in HT-29. (**B**,**F**) Cell cycle phase distribution and quantification results in MDA-MB-231. (**C**,**G**) Cell cycle phase distribution and quantification results in U251. (**D**,**H**) Cell cycle phase distribution and quantification results in Panc-1R. The results for the percentage of cells in G1, S and G2/M phases are shown as means and S.D. Two way-ANOVA with a post hoc Bonferroni–Dunn method test showing *p* values for comparison between control and treatment: (ns) denotes non-significant, (***) denotes *p* < 0.001 and (****) denotes *p* < 0.0001.

To further elucidate whether the synergism of rhArg + ABT263 was due to other factors, we monitored the expression of CDK2 and cyclin A, two important cell cycle regulators that control S-phase cell cycle progression and also cell cycle distribution. Cell cycle analysis revealed that rhArg alone induced S-phase arrest, increasing the S-phase population by 7–13% in HT-29 (Figure 4A,E), MDA-MB-231 (Figure 4B,F) and U251 (Figure 4C,G) respectively. rhArg alone induced G2/M-phase arrest by increasing the G2/M-phase population by 8% in Panc-1R (Figure 4D,H). A amount of 2–4 µM of ABT263 was non-cytotoxic, except in MDA-MB-231. Therefore, the cell cycle distribution of ABT263 alone in HT-29, U251 and Panc-1R was comparable to that of the non-treatment control (Figure 4). Remarkably, rhArg + ABT263 further elicited S-phase arrest in all cancer cells. After combination treatment, we observed a drop in G1-phase cell population with a concomitant increase in S-phase cell population. The percentages of S-phase cell population were 39% in HT-29 (CTL: 24%); 67% in MDA-MB-231 (CTL: 35%); 35% in U251 (CTL: 16%) and 49% in Panc-1R (CTL: 48%) (Table 3). For the case of Panc-1R, despite the comparable S-phase distribution between the control and combination treatment, we observed that this percentage (49%) was significantly higher than that of rhArg alone (43%) (Table 3). The combination, therefore, induced a minor S-phase and major G2/M-phase cell cycle arrest in Panc-1R.

### 3.5. rhArg + ABT263 Outperformed Other Amino Acid Depletion Agents Combined with ABT263

**Figure 5:**
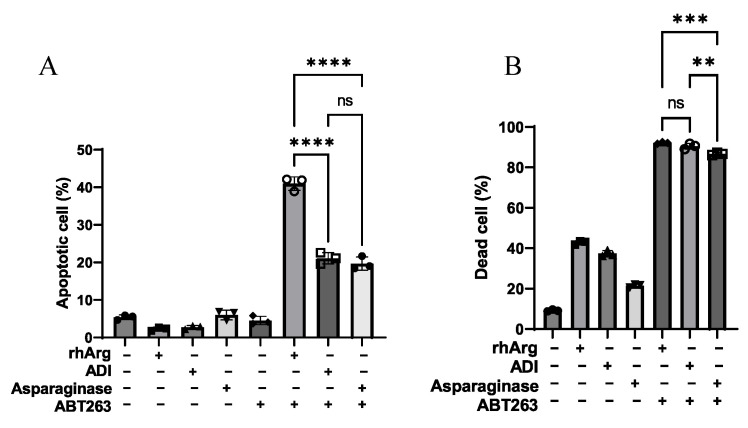

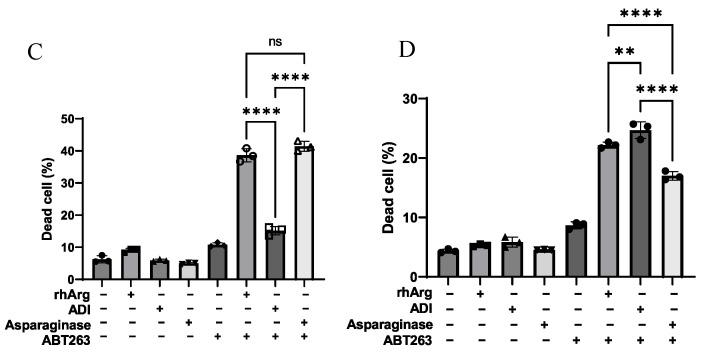
Drug comparative study using annexin V/PI assay to study the cytotoxic effects. Apoptosis or total dead cells in (**A**) HT-29, (**B**) MDA-MB-231, (**C**) U251 and (**D**) Panc-1R cells after 3 days’ treatment with control, 2 U/mL rhArg, 5 µg/mL asparaginase, 10 µg/mL ADI, 2 µM ABT263 alone, 4 µM ABT263 alone (Panc-1R only) or their combinations. Apoptotic cells = early apoptotic cells + late apoptotic cells. Dead cells = necrotic cells + apoptotic cells. Statistical analysis was carried out by one-way ANOVA with a post hoc Tukey test (*n* = 3). (ns) denotes non-significant, (**) denotes *p* < 0.01, (***) denotes *p* < 0.001 and (****) denotes *p* < 0.0001. The raw representative dot plot is shown in Supplementary Figure S6.

To determine whether rhArg was the most favorable amino acid depletion agent to be combined with ABT263, we compared rhArg to two additional amino acid depletion agents, ADI and asparaginase. ABT263 was combined with ADI (10 µg/mL) or asparaginase (5 µg/mL) to evaluate the cytotoxic effect. Resistance to ADI was observed in U251; however, further dose escalation was deemed unlikely to overcome this resistance. The concentrations used were substantially above the IC_50_ values reported in the literature and listed in Table 1 [27]. As depicted in Figure 5, the quantification of dead cells in HT-29 was omitted, as the level of necrosis was comparable across all treatment groups. In this model, the rhArg + ABT263 combination induced apoptosis in over 40% of cells, which was 2-fold higher than that achieved by ADI or asparaginase combinations. In contrast, ADI + ABT263 and asparaginase + ABT263 demonstrated a similar, lower efficacy (~20%) in HT-29. In MDA-MB-231 cells, rhArg + ABT263 and ADI + ABT263 were equally effective, each inducing comparable cell death (*p* > 0.05). Asparaginase + ABT263 was slightly less potent, resulting in 5% fewer dead cells. U251 cells were completely resistant to ADI monotherapy (Table 1), which explained the significantly lower cell death with ADI + ABT263 compared to rhArg + ABT263. However, the total dead cell counts for rhArg + ABT263 and asparaginase + ABT263 were comparable (*p* > 0.05, Figure 5). In Panc-1R cells, the overall cell death for rhArg + ABT263 and ADI + ABT263 was similar. Asparaginase + ABT263 elicited only minimal cell death (~17%). Notably, the rhArg + ABT263 combination was the only one that demonstrated significant activity across all four cancer cell lines. These findings suggested that the rhArg + ABT263 combination represented an applicable therapeutic strategy against the four in vitro models.

### 3.6. rhArg + ABT263 Produced the Most Pronounced Synergistic Effect with Elevated Apoptotic Markers and Downregulated the Expression of Cell Cycle Regulators

To confirm that the cytotoxic effect was contributed by apoptosis, cleaved caspase 3 and cleaved PARP were selected as apoptosis markers in this study [28,29,30]. In addition, based on the observed S-phase accumulation following combination treatment in Table 3, we also analyzed the expression of cyclin A and CDK2, as they regulate S-phase progression [31].

**Figure 6:**
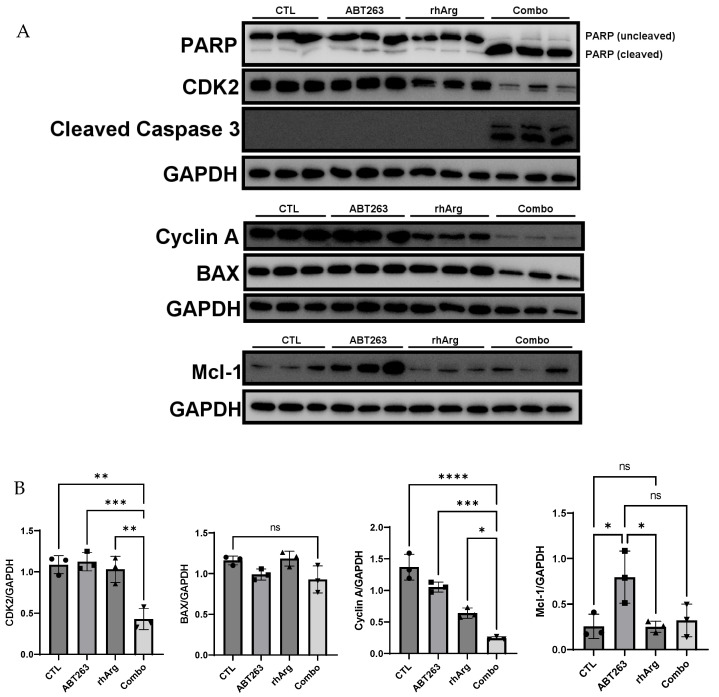
Western blot and corresponding densitometry analyses of protein expression in HT-29. (**A**) Western blot analysis of PARP, cyclin A, CDK2, cleaved caspase 3, BAX and Mcl-1 protein expressions in HT-29 cells after 48 h incubation with 2 U/mL rhArg, 2 μM ABT263 or their combination before cell harvest. Gel images with three independent experiments are shown. (**B**) Summary of protein quantification results are expressed as means ± standard deviation (*n* = 3). One way-ANOVA with a post hoc Tukey test showing *p* values for comparison between the control and treatment group: ns: non-significant, * *p* < 0.05, ** *p* < 0.01, *** *p* < 0.001 and **** *p* < 0.0001.

**Figure 7:**
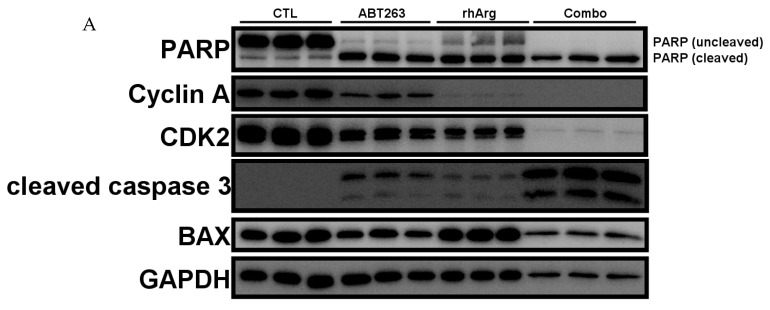

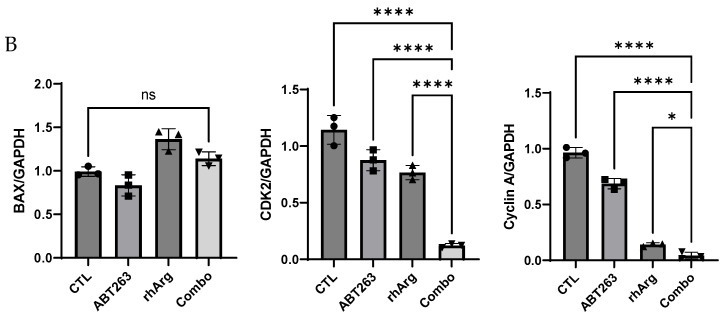
Western blot and corresponding densitometry analyses of protein expression in MDA-MB-231. (**A**) Western blot analysis of PARP, cyclin A, CDK2, cleaved caspase 3 and BAX protein expressions in MDA-MB-231 cells after 48 h incubation with 2 U/mL rhArg, 2 μM ABT263 or their combination before cell harvest. Gel images with three independent experiments are shown. (**B**) Summary of protein quantification results are expressed as means ± standard deviation (*n* = 3). One way-ANOVA with a post hoc Tukey test showing *p* values for comparison between the control and treatment group: ns: non-significant, * *p* < 0.05, and **** *p* < 0.0001.

**Figure 8:**
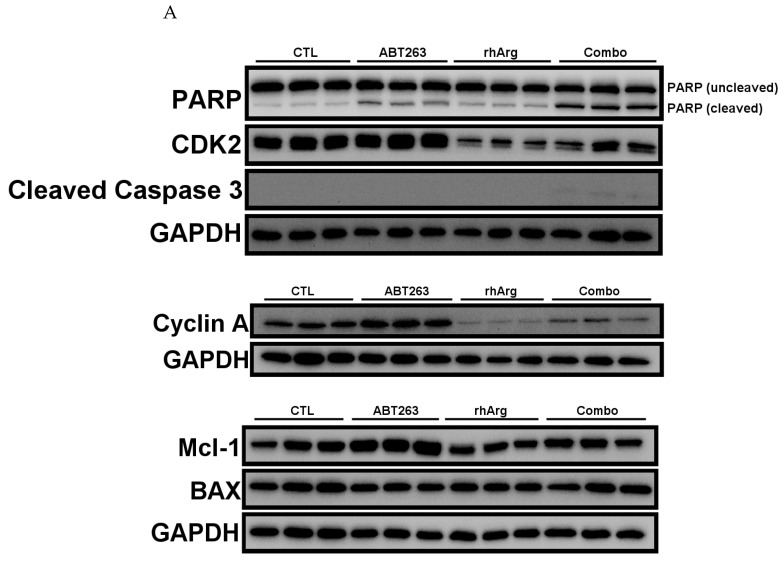

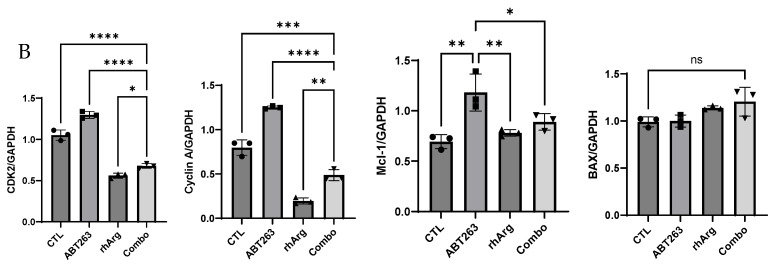
Western blot and corresponding densitometry analyses of protein expression in U251. (**A**) Western blot analysis of PARP, cyclin A, CDK2, cleaved caspase 3 and BAX protein expressions in U251 cells after 48 h incubation with 2 U/mL rhArg, 2 μM ABT263 or their combination before cell harvest. Gel images with three independent experiments are shown. (**B**) Summary of protein quantification results are expressed as means ± standard deviation (*n* = 3). One way-ANOVA with a post hoc Tukey test showing *p* values for comparison between the control and treatment group: ns: non-significant, * *p* < 0.05, ** *p* < 0.01, *** *p* < 0.001 and **** *p* < 0.0001.

**Figure 9:**
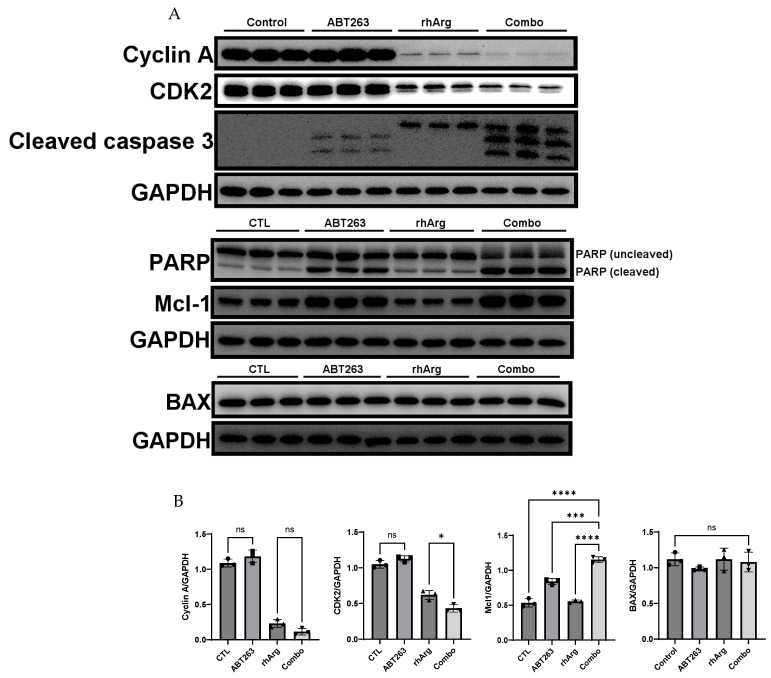
Western blot and corresponding densitometry analyses of protein expression in Panc-1R. (**A**) Western blot analysis of PARP, cyclin A, CDK2, cleaved caspase 3 and BAX protein expressions in Panc-1R cells after 48 h incubation with 2 U/mL rhArg, 4 μM ABT263 or their combination before cell harvest. Gel images with three independent experiments are shown. (**B**) Summary of protein quantification results are expressed as means ± standard deviation (*n* = 3). One way-ANOVA with a post hoc Tukey test showing *p* values for comparison between the control and treatment group: ns: non-significant, * *p* < 0.05, *** *p* < 0.001 and **** *p* < 0.0001.

Remarkably, we observed a general increase in both cleaved caspase 3 and cleaved PARP across all in vitro models (Figure 6, Figure 7, Figure 8 and Figure 9). Although the expression of cleaved caspase 3 was relatively weak in U251 (Figure 8), we still observed a significant increase in cleaved PARP. This confirmed that the cell death was likely due to apoptosis.

In agreement with the cell cycle analysis in the previous section, the expression levels of CDK2 and cyclin A were downregulated by the combination treatment in HT-29 (Figure 6), MDA-MB-231 (Figure 7) and Panc-1R (Figure 9). Although the downregulation of both CDK2 and cyclin A in Panc-1R treated with the combination was marginal (Figure 9B), we were able to observe the trend. Interestingly, ABT263 alone upregulated both CDK2 and cyclin A expression in U251 (Figure 8); therefore, the higher expression of both proteins in the combination group compared to that of rhArg alone was possibly due to ABT263, and rhArg abrogated this phenomenon. These results indicated that the synergistic effect was mediated not only by apoptosis but also possibly by cell cycle arrest.

To further evaluate the role of other anti-apoptotic proteins, we measured the expression of Mcl-1, a key factor that mediates resistance to ABT263 [32,33]. Treatment with ABT263 alone significantly upregulated Mcl-1 expression in HT-29 (Figure 6B), U251 (Figure 8B) and Panc-1R (Figure 9B), signifying that 48 h ABT263 treatment was able to trigger drug resistance mechanisms in cancer cells. Because MDA-MB-231 was the most sensitive cell line to the combination (around 80% apoptosis), we did not measure Mcl-1 expression in MDA-MB-231. rhArg alone did not modulate the expression of Mcl-1, but the combination inhibited ABT263-mediated Mcl-1 upregulation in HT-29 (Figure 6B) and U251 (Figure 8B) and maintained Mcl-1 expression at basal levels in these two cell lines. The relative resistance of Panc-1R to the combination treatment (20% apoptosis) can be partly explained by its compensatory upregulation of Mcl-1, which was further triggered by the combination (Figure 9B). We also monitored the expression of a pro-apoptotic protein BAX; however, BAX levels were comparable between the combination group and the control group (Figure 6, Figure 7, Figure 8 and Figure 9).

## 4. Discussion

The four in vitro models employed in this study presented distinct therapeutic hurdles. Chemotherapy remains the mainstay of treatment for these cancers. However, successful chemotherapeutic treatment is compromised by drug resistance and subsequent relapse. Mutations in key signaling molecules and aberrant pathway activation, both common in cancer, mediate poor responses to chemotherapy. Arginine depletion is an emerging therapeutic approach that selectively targets those malignant cells which harbor a defective urea cycle. The positive phase III results in treating unresectable pleural mesothelioma by Pegargiminase further encourages the development of arginine depletion agents. Our group has focused on the development of rhArg in the past decade, and rhArg was proven to be effective in ADI-resistant U251 as depicted in Table 1 [8]. This result agreed with the reported literature as U251 was ASS-positive [34]. Moreover, we found that arginine starvation induced by rhArg exhibits high sensitivity in cancer cell lines due to their highly proliferative nature. However, in less-proliferative cell models, such as 3T3-L1 differentiated adipocytes and SH-SY5Y differentiated neurons, rhArg demonstrates non-toxicity (Supplementary Figure S7). This suggests that rhArg could serve as a promising platform to combine with various cytotoxic drugs as an adjunct treatment against cancer.

In this study, ABT263 and metformin were evaluated and their combinations with rhArg were tested in the four in vitro models. rhArg + metformin was synergistic in a time-dependent manner which abrogated the colony formation ability of HT-29. ABT263 was found to produce the most pronounced synergistic anticancer effect when combined with rhArg. Only after combination treatment were apoptotic markers upregulated. This combination pair was effective in all in vitro models, with CI values < 1 (Table 2). ABT263 is an investigational drug, but a phase II study found ABT263 monotherapy ineffective in SCLC patients, strongly recommending future combinatorial regimens (NCT00445198) [35]. Remarkably, our combinatorial study potentiated the cytotoxic effect with a corresponding reduction in drug dosages, as evidenced by the DRI values in Table 2. Based on a phase I clinical trial evaluating the pharmacokinetics and pharmacodynamics of ABT263 in SCLC patients, doses ≥ 225 mg achieved minimum plasma exposure predicted to be within the therapeutic range, with the 225 mg dose yielding a Cmax of 5.2 μM [36]. Efficacy of rhArg + ABT263 was corroborated by the comparative study depicted in Figure 5. The rhArg + ABT263 combination demonstrated superior cytotoxicity compared to other amino acid depletion agents, further supporting rhArg as the preferred candidate for use with Bcl-2 inhibitors. Based on the results of this study and the reported synergy between ADI-PEG20 and Bcl-xL inhibitor in multiple ASS-negative cancer cell lines [37], we proposed that the therapeutic application of the rhArg + ABT263 combination could be broadened to include multiple cancer types. The concentrations of ABT263 (2–4 µM) required for the observed combination-induced cytotoxicity are clinically achievable, underscoring the translational potential of our findings.

A previous study revealed that arginine depletion triggered Bcl-2 expression in hepatocellular carcinoma cell lines as a survival strategy [22]. Although we did not directly monitor Bcl-2 expression, we anticipated that cancer cells evaded rhArg monotherapy partly due to Bcl-2 upregulation. Previous reports revealed that elevated Bcl-2 expression would have sensitized cancer cells to ABT263 treatment [38,39]. Therefore, ABT263 was employed to inhibit this rescue mechanism, thereby enhancing the efficacy of rhArg treatment.

ABT263 was reported to induce Mcl-1 expression in multiple cancer types [40,41]. rhArg was able to tackle the potential drug resistance issue caused by ABT263 monotherapy due to Mcl-1 upregulation (Figure 6 and Figure 8), which meant that the combination was likely to circumvent one of the drug resistance pathways. The gemcitabine-resistant Panc-1R cell line exhibited a reduced susceptibility to apoptosis compared to the wild-type cells, which corresponded to the least sensitive phenotype to the combination among the four models (Figure 3F) [42]. The combination of rhArg with ABT199 did not induce cell death, even though it was synergistic as described in Table 2, indicating that single inhibition of Bcl-2 was not enough to incur a significant cytotoxic effect and the involvement of other anti-apoptotic proteins might protect cancer cells from apoptosis. A 3-day apoptotic assay suggested that rhArg + ABT199 was likely to be cytostatic. We acknowledge that Bcl-xL, rather than Bcl-2, is the main anti-apoptotic protein that protected cells from apoptosis under arginine depletion [37]. However, there is currently no Bcl-xL inhibitor that has entered clinical trials or possesses an established toxicity profile. We anticipate that developing the rhArg + ABT263 combination pair would be more time- and cost-effective.

A previous report revealed the regulatory role of CDK2 on Mcl-1 expression [37]. A concomitant decrease in Mcl-1 occurred alongside CDK2 downregulation [37]. Together with the fact that rhArg induced S-phase arrest, we also monitored CDK2 expression in our study. Although rhArg alone downregulated CDK2 expression in both U251 and Panc-1R cells, Mcl-1 expression did not decrease below the levels of the control group (Figure 8 and Figure 9). Mcl-1 expression was further promoted by the combination despite the lowest CDK2 expression in Panc-1R cells (Figure 9). These results suggested that Mcl-1 expression was not solely dependent on CDK2. However, we did not rule out a regulatory role for CDK2 on Mcl-1, as it was also possible that other uncharacterized mechanism(s) participated in maintaining basal Mcl-1 expression.

Bax is a pro-apoptotic protein that is responsible for inducing mitochondrial outer membrane permeabilization (MOMP) to execute cell death. Nevertheless, we did not observe any change in this protein across all models. Our results agreed with what had been reported, as we only measured the cytosolic Bax expression [37]. Following the sequestration of anti-apoptotic proteins by ABT263, free Bax induces MOMP at the mitochondrial surface. Consistent with this mechanism, Bax upregulation was only observed in the mitochondrial fraction [37].

We recognize there are several weaknesses in this study: (1) We did not address the inconsistency of Mcl-1 expression compared to the other reported literature and the participation of other anti-/pro-apoptotic proteins in inducing cell death. (2) The DRI values suggested that the rhArg dose could have been reduced by at least 2-fold, but in this study, we used 2 U/mL (10 µg/mL) rhArg. (3) rhArg alone and in combination both induced cell cycle arrest, but the percentage change in cell cycle distribution did not fully address the observed growth inhibition (Table 3). The aim of this study was to provide proof-of-concept for identifying potential combination partners for rhArg. We did not consider that 2 U/mL was an optimal dose in combination. In addition, other uncharacterized mechanisms (e.g., autophagy) might have contributed to the synergism. (4) The medium we used in this study contained the supplement GlutaMAX™, which is a dipeptide formed by alanine and glutamine. GlutaMAX™ enabled a gradual release of glutamine and alanine but was itself not a direct target of asparaginase. As asparaginase was reported to deplete both asparagine and glutamine, we did not rule out the fact that the lower efficacy of asparaginase + ABT263 compared to rhArg + ABT263 was due to the incapability of asparaginase to deplete GlutaMAX™, meaning that asparaginase in our experimental conditions depleted asparagine only. (5) We did not measure the expression levels of Bcl-2 and Bcl-xL after the drug treatment despite focusing on the Bcl-2 class inhibitors. The interaction between the anti-apoptotic proteins might further explain the sensitivity of different cell lines to the combination treatment. (6) An in vivo study is warranted to study the combination efficacy as well as the potential thrombocytopenia effect. We anticipate that the above issues could be addressed in the future studies.

## 5. Conclusions

In summary, our study discovered a synergistic combination with broad-spectrum anticancer effects. The combination of rhArg and ABT263 greatly enhanced the cytotoxic effect and cell cycle arrest, signifying that ABT263 was a favorable partner to combine with. The cytotoxic effect was likely due to apoptosis as evidenced by elevated apoptotic markers. rhArg possibly circumvented Bcl-2 inhibitor drug resistance mechanisms by modulating the expression of Mcl-1. Further studies are warranted to establish the underlying mechanisms, the dose-dependent role of rhArg in combination-induced apoptosis and the in vivo validation of the combination efficacy. The findings provide valuable insights into the rhArg-ABT263 combination in multiple in vitro models.

## Figures and Tables

**Table 1:** Determination of IC_50_ values of drug candidates in HT-29, MDA-MB-231, U251 and Panc-1R.

Drugs	IC_50_ µg/mL ± SD (µM)			
	HT-29	MDA-MB-231	U251	Panc-1R
GCN2iB	7.47 ± 3.45 (16.53)	39.69 ± 26.04 (87.84)	21.40 ± 7.81 (47.36)	23.11 ± 2.06 (51.15)
ABT263	11.64 ± 0.77 (11.94)	2.14 ± 0.30 (2.20)	9.22 ± 1.61 (9.46)	14.60 ± 0.51 (14.98)
Metformin	5634.00 ± 378.00 (17,842.7)	2967.00 ± 216.00 (9396.4)	ND	ND
ABT199	20.93 ± 1.13 (24.10)	14.70 ± 1.97 (16.93)	13.61 ± 1.11 (15.67)	ND
IC_50_ of arginine depletion agents				
rhArg (µg/mL ± SD (U/mL))				
ADI (µg/mL ± SD)				
rhArg	0.32 ± 0.09 (0.062)	0.24 ± 0.12 (0.047)	0.96 ± 0.40 (0.186)	0.21 ± 0.05 (0.041)
ADI	0.00830 ± 0.0017	0.0071 ± 0.0015	Resistant	0.0085 ± 0.0026

The cells were incubated with compounds for 72 h. IC_50_ values shown (±SD) are derived from at least three independent replicate experiments. ND: not determined.

**Table 2:** Evaluation of the combination index (CI) and dose reduction index (DRI) for the combined treatment of rhArg with various chemical entities in HT-29, MDA-MB-231, U251 and Panc-1R cells.

Cell Lines	Chemical Entities	Treatment Time	CI	DRI	
				rhArg	Chemical Entities
HT-29	Metformin	48 h	1.27 ± 0.21	1.73 ± 0.27	1.52 ± 0.23
		96 h	0.65 ± 0.03	3.82 ± 0.17	2.58 ± 0.12
	ABT263	72 h	0.71 ± 0.03	3.03 ± 0.11	2.64 ± 0.10
	ABT199	72 h	0.69 ± 0.04	2.99 ± 0.11	2.72 ± 0.10
MDA-MB-231	Metformin	48 h	1.91 ± 0.46	1.16 ± 0.31	1.07 ± 0.29
		96 h	0.84 ± 0.07	2.68 ± 0.21	2.15 ± 0.17
	ABT263	72 h	0.90 ± 0.03	2.31 ± 0.07	2.16 ± 0.07
	ABT199	72 h	0.88 ± 0.06	2.62 ± 0.16	2.02 ± 0.12
U251	ABT199	72 h	0.82 ± 0.04	2.52 ± 0.13	2.40 ± 0.12
	ABT263	72 h	0.47 ± 0.02	4.13 ± 0.16	4.33 ± 0.16
Panc-1R	ABT263	72 h	0.90 ± 0.02	1.60 ± 0.04	3.71 ± 0.10

Data are presented as mean ± standard deviation with 3 independent experiments were performed. CI < 1, =1 and >1 suggest synergism, additivity and antagonism, respectively.

**Table 3:** Cell cycle distribution of cells after 48 h treatment as depicted in Figure 4.

HT-29	G1 (%)	S (%)	G2/M (%)
Control	63.03 ± 0.55	23.77 ± 1.07	13.21 ± 0.52
rhArg	48.10 ± 0.96	30.94 ± 1.83	20.96 ± 2.58
ABT263	62.48 ± 0.10	26.19 ± 2.06	11.33 ± 2.03
Combo	47.92 ± 0.20	39.17 ± 1.52	12.90 ± 1.33
MDA-MB-231	G1 (%)	S (%)	G2/M (%)
Control	49.65 ± 0.92	35.32 ± 2.58	15.03 ± 1.70
rhArg	45.59 ± 1.10	47.03 ± 1.51	7.38 ± 0.43
ABT263	28.63 ± 0.17	60.65 ± 1.30	10.72 ± 1.31
Combo	28.92 ± 1.60	67.41 ± 1.15	3.67 ± 0.68
U251	G1 (%)	S (%)	G2/M (%)
Control	48.76 ± 0.95	16.19 ± 0.41	35.10 ± 0.96
rhArg	39.60 ± 0.99	30.14 ± 0.63	30.25 ± 1.58
ABT263	44.23 ± 0.86	21.50 ± 0.69	34.26 ± 0.65
Combo	36.26 ± 1.69	35.21 ± 1.51	28.54 ± 0.34
Panc-1R	G1 (%)	S (%)	G2/M (%)
Control	46.83 ± 1.12	47.77 ± 1.26	5.39 ± 0.32
rhArg	43.85 ± 0.71	42.91 ± 0.82	13.24 ± 0.26
ABT263	47.42 ± 1.06	46.67 ± 1.11	5.90 ± 0.17
Combo	39.98 ± 0.70	48.76 ± 0.78	11.23 ± 0.37

## Data Availability

The original contributions presented in this study are included in the article/Supplementary Materials. Further inquiries can be directed to the corresponding author(s).

## Supplementary Materials

The following supporting information can be downloaded at https://www.mdpi.com/article/10.3390/cells15020164/s1, Figure S1: Arginine level changes in cell culture medium; Figure S2: Representative dot plot image of Annexin V/PI assessment of apoptosis for Figure 1 in HT-29 cells; Figure S3: Representative dot plot image of Annexin V/PI assessment of apoptosis for Figure 2 in MDA-MB-231 cells; Figure S4: Representative dot plot image of Annexin V/PI assessment of apoptosis for Figure 3 in U251 and Panc-1R cells; Figure S5: Colony-forming assay for HT-29 after different treatments; Figure S6: Representative dot plot image of Annexin V/PI assessment of apoptosis for Figure 5. Figure S7: Cell viability of 3T3-L1 and SYSH5Y cells was unaffected under rhArg treatment. File S1. Original Images for Blot.

## Abbreviations

The following abbreviations are used in this manuscript:

rhArg	Recombinant human arginase
PDAC	Pancreatic ductal carcinoma
TNBC	Triple-negative breast cancer
CRC	Colorectal cancer
