# Supplementary material for: The Synergistic Effects of rhArg with Bcl-2 Inhibitors or Metformin Co-Treatment in Multiple Cancer Cell Models
Source: Cells. 2026 Jan 16;15(2):164. doi: 10.3390/cells15020164 (PMC12839320; doi:10.3390/cells15020164)
Supplement: Supplementary file 1 [file cells-15-00164-s001.zip › cells-4044689 Supplementary Figure (Final).pptx]

## Slide 1
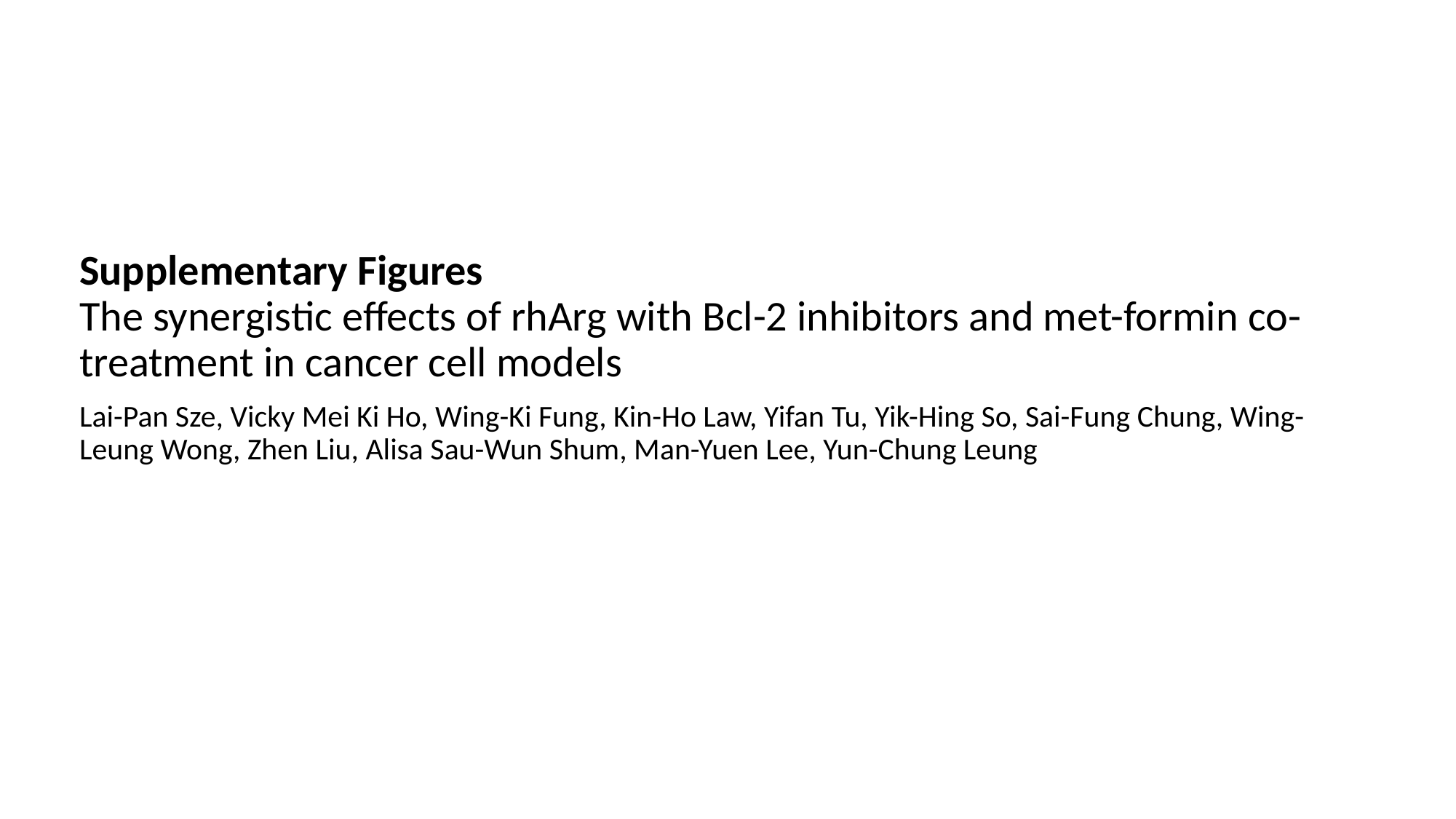

Supplementary Figures
The synergistic effects of rhArg with Bcl-2 inhibitors and met-formin co-treatment in cancer cell models
Lai-Pan Sze, Vicky Mei Ki Ho, Wing-Ki Fung, Kin-Ho Law, Yifan Tu, Yik-Hing So, Sai-Fung Chung, Wing-Leung Wong, Zhen Liu, Alisa Sau-Wun Shum, Man-Yuen Lee, Yun-Chung Leung

## Slide 2
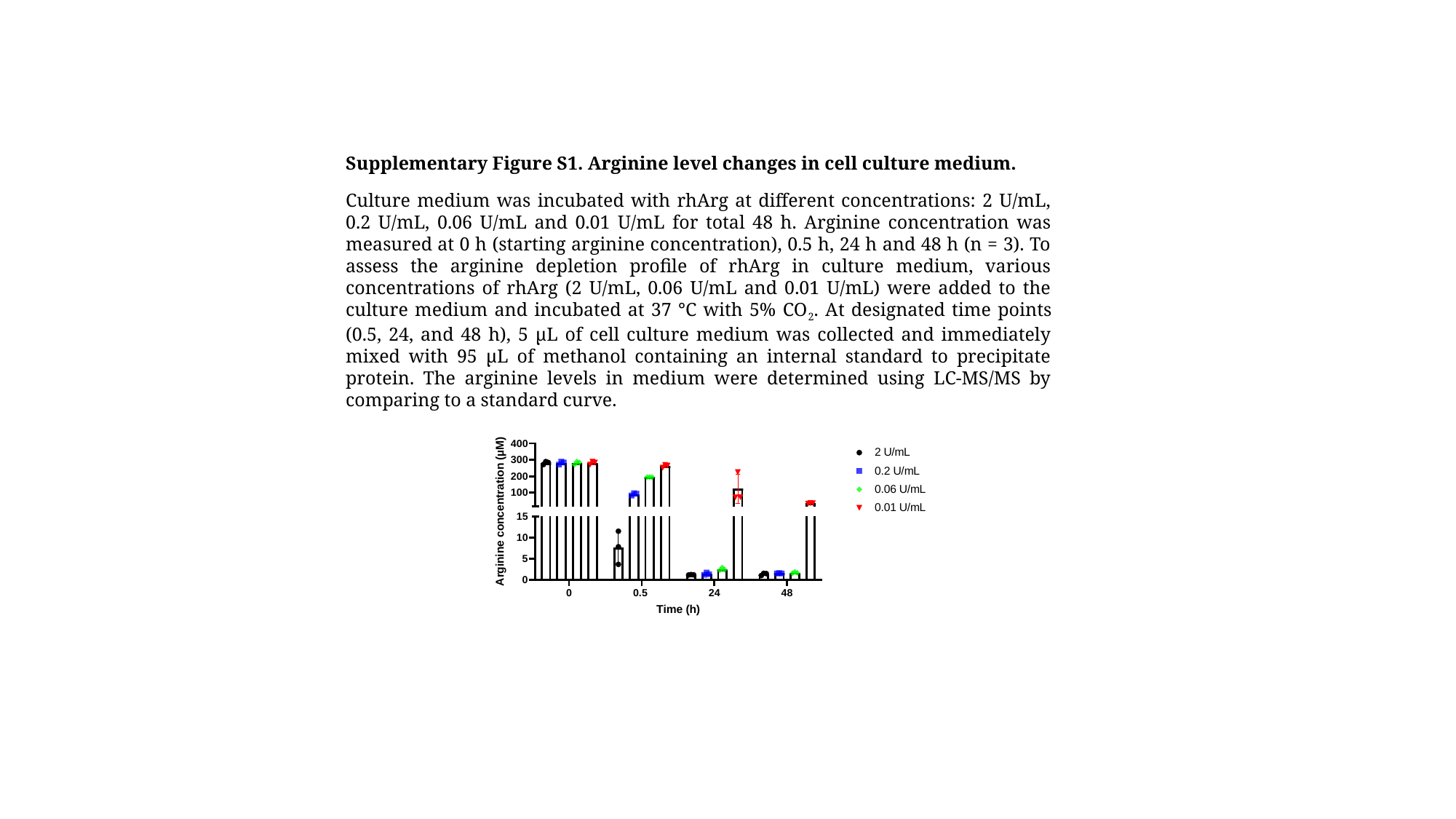

Supplementary Figure S1. Arginine level changes in cell culture medium.
Culture medium was incubated with rhArg at different concentrations: 2 U/mL, 0.2 U/mL, 0.06 U/mL and 0.01 U/mL for total 48 h. Arginine concentration was measured at 0 h (starting arginine concentration), 0.5 h, 24 h and 48 h (n = 3). To assess the arginine depletion profile of rhArg in culture medium, various concentrations of rhArg (2 U/mL, 0.06 U/mL and 0.01 U/mL) were added to the culture medium and incubated at 37 °C with 5% CO2. At designated time points (0.5, 24, and 48 h), 5 µL of cell culture medium was collected and immediately mixed with 95 µL of methanol containing an internal standard to precipitate protein. The arginine levels in medium were determined using LC-MS/MS by comparing to a standard curve.

## Slide 3
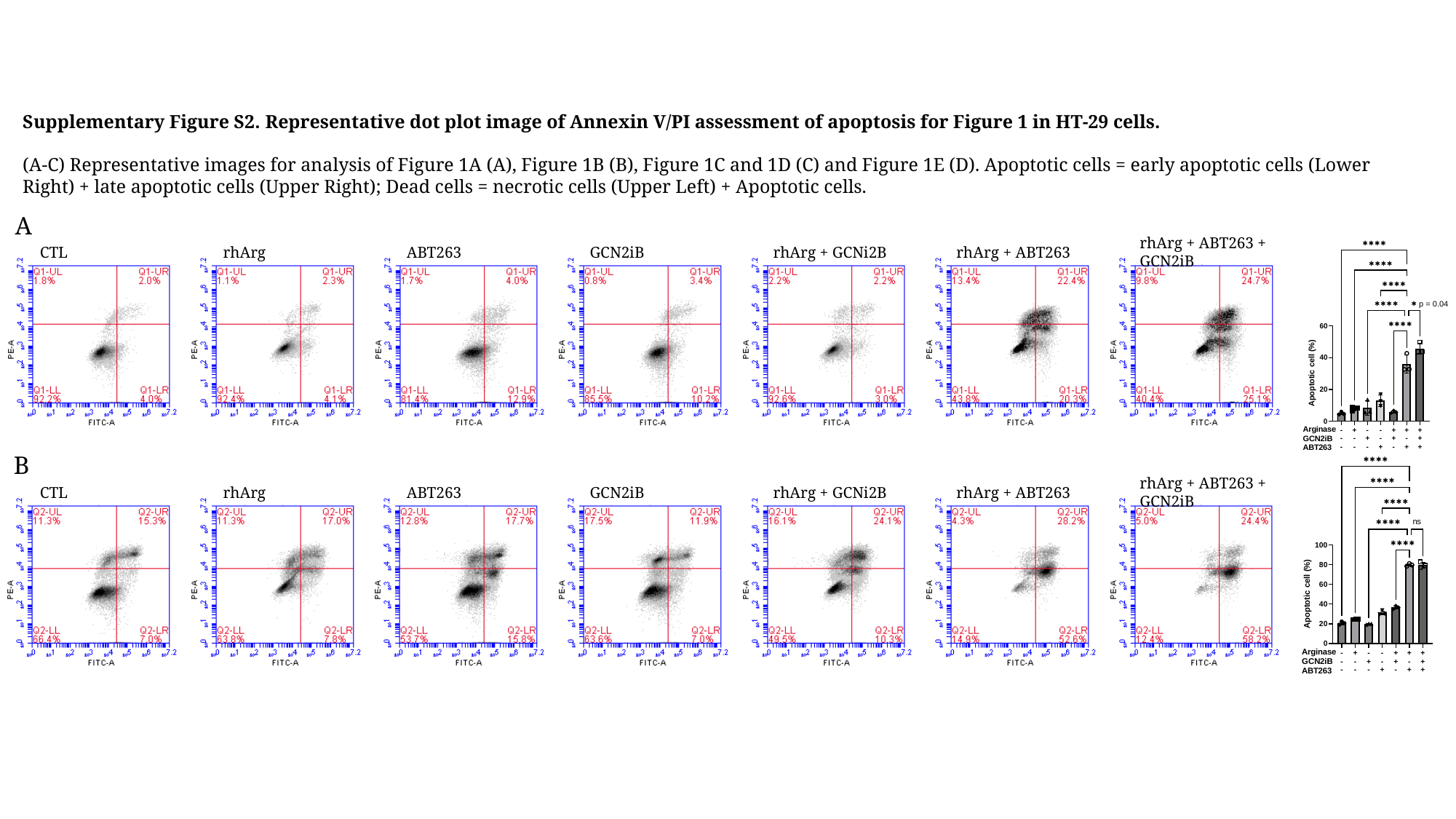

Supplementary Figure S2. Representative dot plot image of Annexin V/PI assessment of apoptosis for Figure 1 in HT-29 cells.
(A-C) Representative images for analysis of Figure 1A (A), Figure 1B (B), Figure 1C and 1D (C) and Figure 1E (D). Apoptotic cells = early apoptotic cells (Lower Right) + late apoptotic cells (Upper Right); Dead cells = necrotic cells (Upper Left) + Apoptotic cells.
A
rhArg + ABT263 + GCN2iB
CTL
rhArg
ABT263
GCN2iB
rhArg + GCNi2B
rhArg + ABT263
B
rhArg + ABT263 + GCN2iB
CTL
rhArg
ABT263
GCN2iB
rhArg + GCNi2B
rhArg + ABT263

## Slide 4
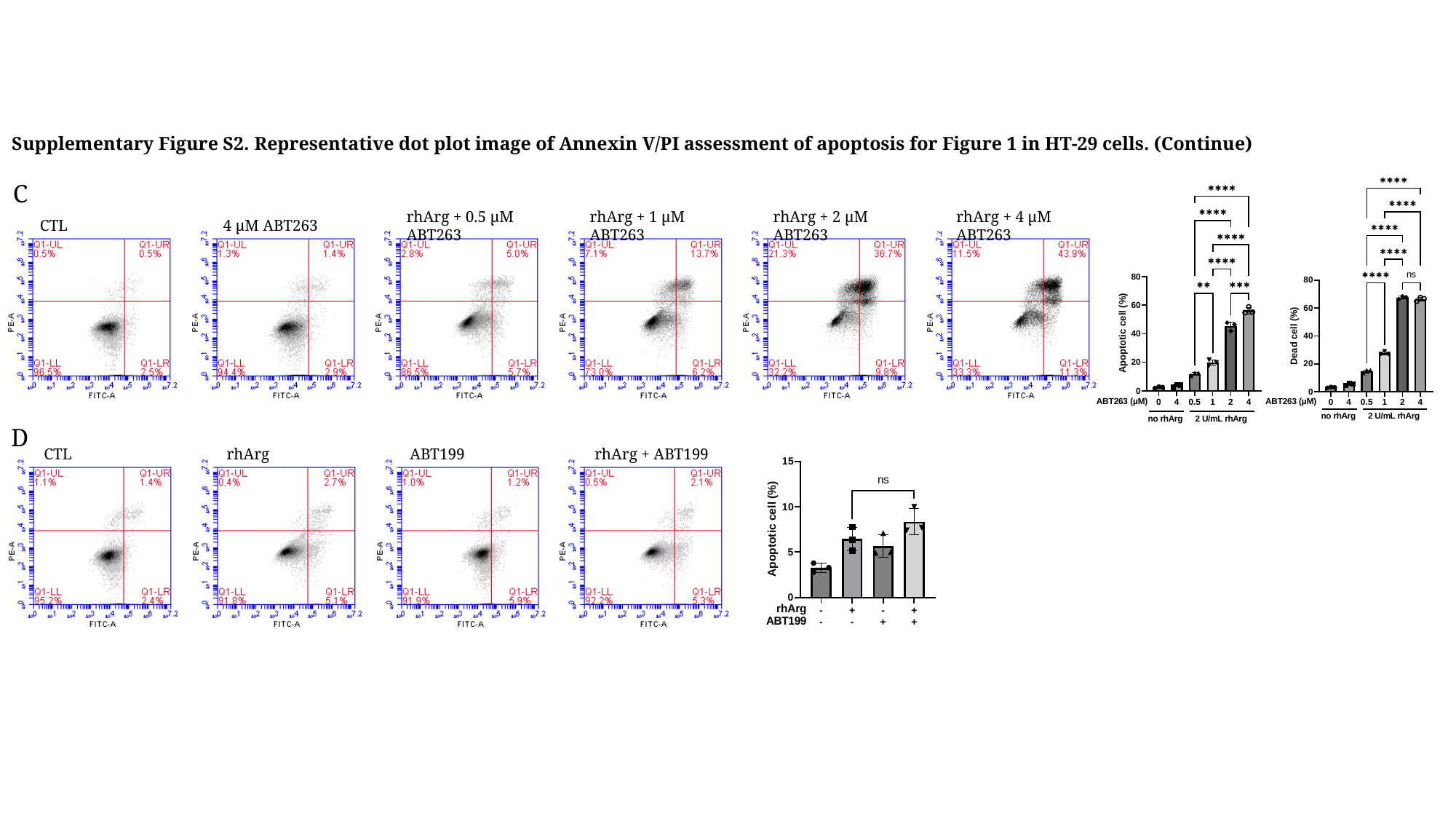

Supplementary Figure S2. Representative dot plot image of Annexin V/PI assessment of apoptosis for Figure 1 in HT-29 cells. (Continue)
C
CTL
4 μM ABT263
rhArg + 0.5 μM ABT263
rhArg + 1 μM ABT263
rhArg + 2 μM ABT263
rhArg + 4 μM ABT263
D
CTL
rhArg
ABT199
rhArg + ABT199

## Slide 5
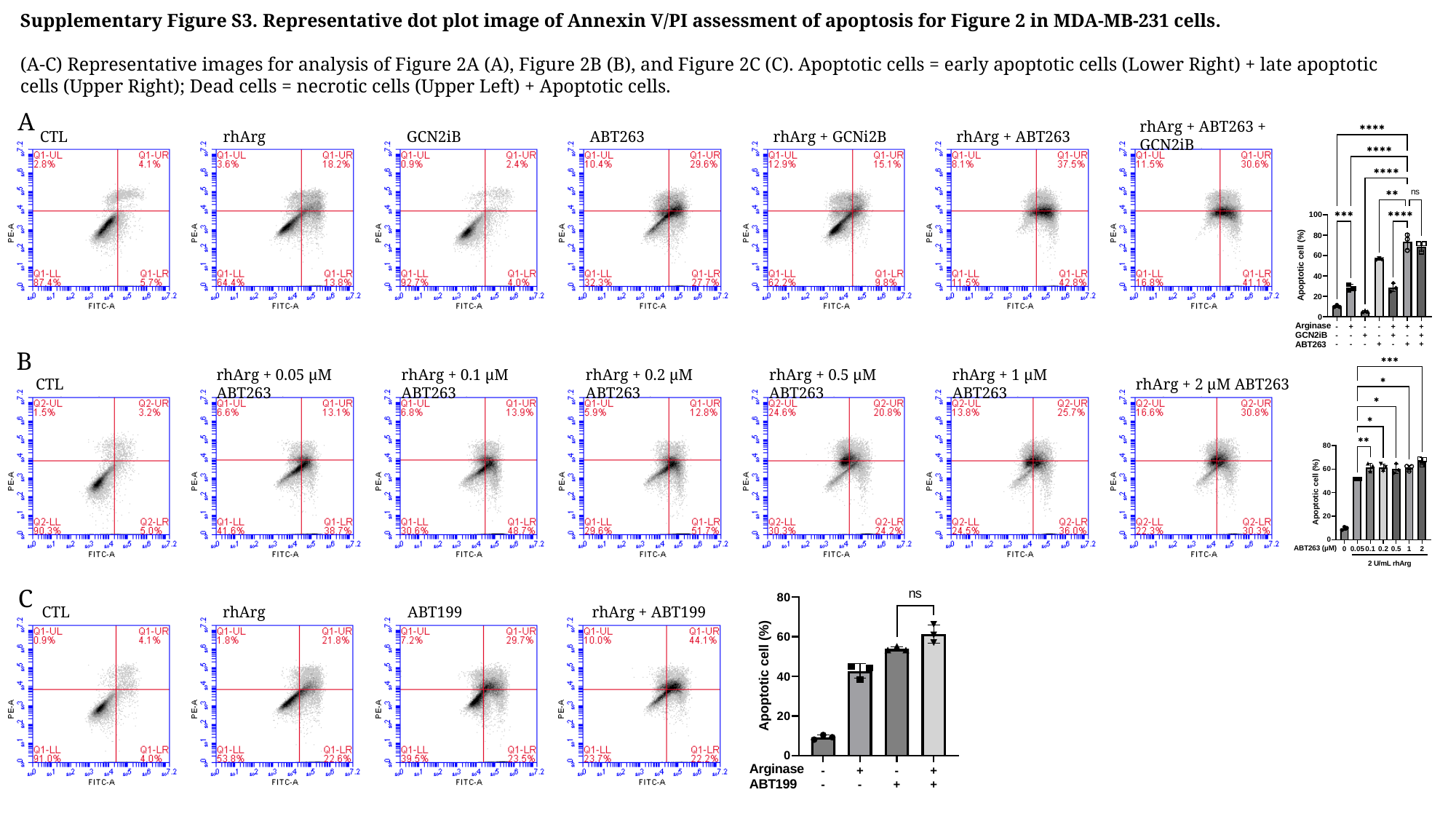

Supplementary Figure S3. Representative dot plot image of Annexin V/PI assessment of apoptosis for Figure 2 in MDA-MB-231 cells.
(A-C) Representative images for analysis of Figure 2A (A), Figure 2B (B), and Figure 2C (C). Apoptotic cells = early apoptotic cells (Lower Right) + late apoptotic cells (Upper Right); Dead cells = necrotic cells (Upper Left) + Apoptotic cells.
A
rhArg + ABT263 + GCN2iB
CTL
rhArg
GCN2iB
ABT263
rhArg + GCNi2B
rhArg + ABT263
B
CTL
rhArg + 0.05 μM ABT263
rhArg + 0.1 μM ABT263
rhArg + 0.2 μM ABT263
rhArg + 0.5 μM ABT263
rhArg + 1 μM ABT263
rhArg + 2 μM ABT263
C
CTL
rhArg
ABT199
rhArg + ABT199

## Slide 6
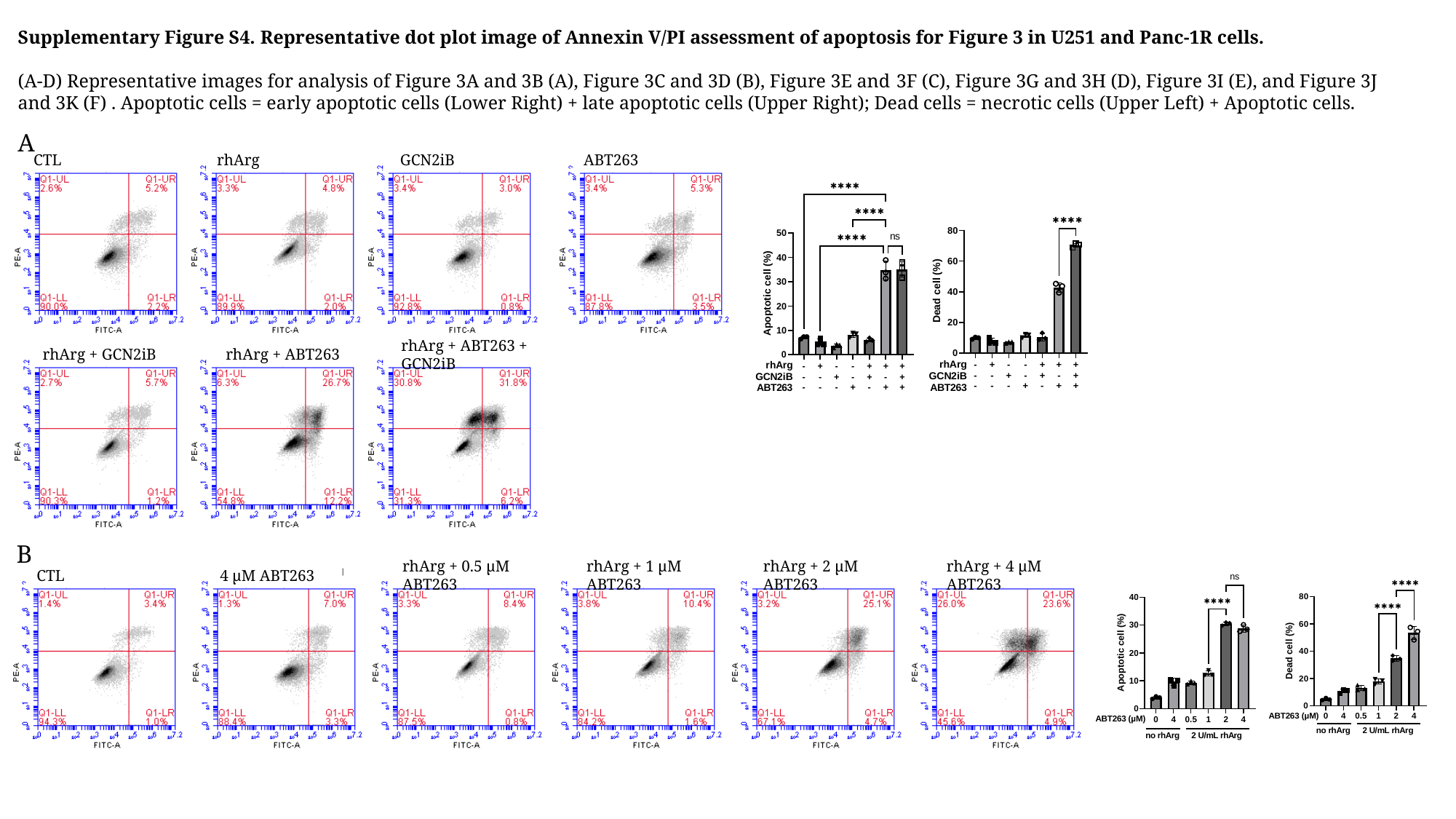

Supplementary Figure S4. Representative dot plot image of Annexin V/PI assessment of apoptosis for Figure 3 in U251 and Panc-1R cells.
(A-D) Representative images for analysis of Figure 3A and 3B (A), Figure 3C and 3D (B), Figure 3E and 3F (C), Figure 3G and 3H (D), Figure 3I (E), and Figure 3J and 3K (F) . Apoptotic cells = early apoptotic cells (Lower Right) + late apoptotic cells (Upper Right); Dead cells = necrotic cells (Upper Left) + Apoptotic cells.
A
CTL
rhArg
GCN2iB
ABT263
rhArg + GCN2iB
rhArg + ABT263
rhArg + ABT263 + GCN2iB
B
CTL
4 μM ABT263
rhArg + 0.5 μM ABT263
rhArg + 1 μM ABT263
rhArg + 2 μM ABT263
rhArg + 4 μM ABT263

## Slide 7
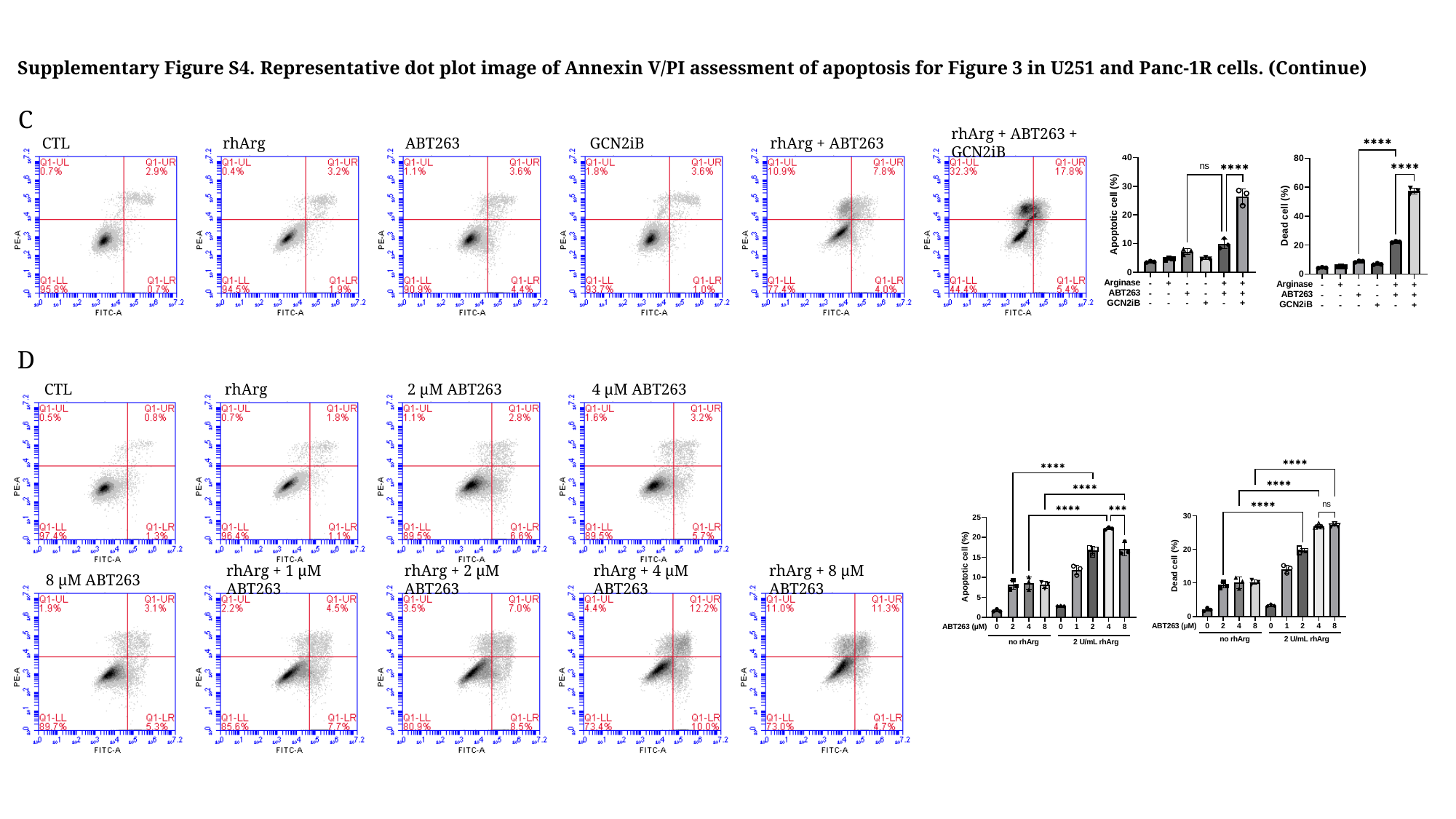

Supplementary Figure S4. Representative dot plot image of Annexin V/PI assessment of apoptosis for Figure 3 in U251 and Panc-1R cells. (Continue)
C
CTL
rhArg
ABT263
GCN2iB
rhArg + ABT263
rhArg + ABT263 + GCN2iB
D
CTL
rhArg
2 μM ABT263
4 μM ABT263
rhArg + 8 μM ABT263
8 μM ABT263
rhArg + 1 μM ABT263
rhArg + 2 μM ABT263
rhArg + 4 μM ABT263

## Slide 8
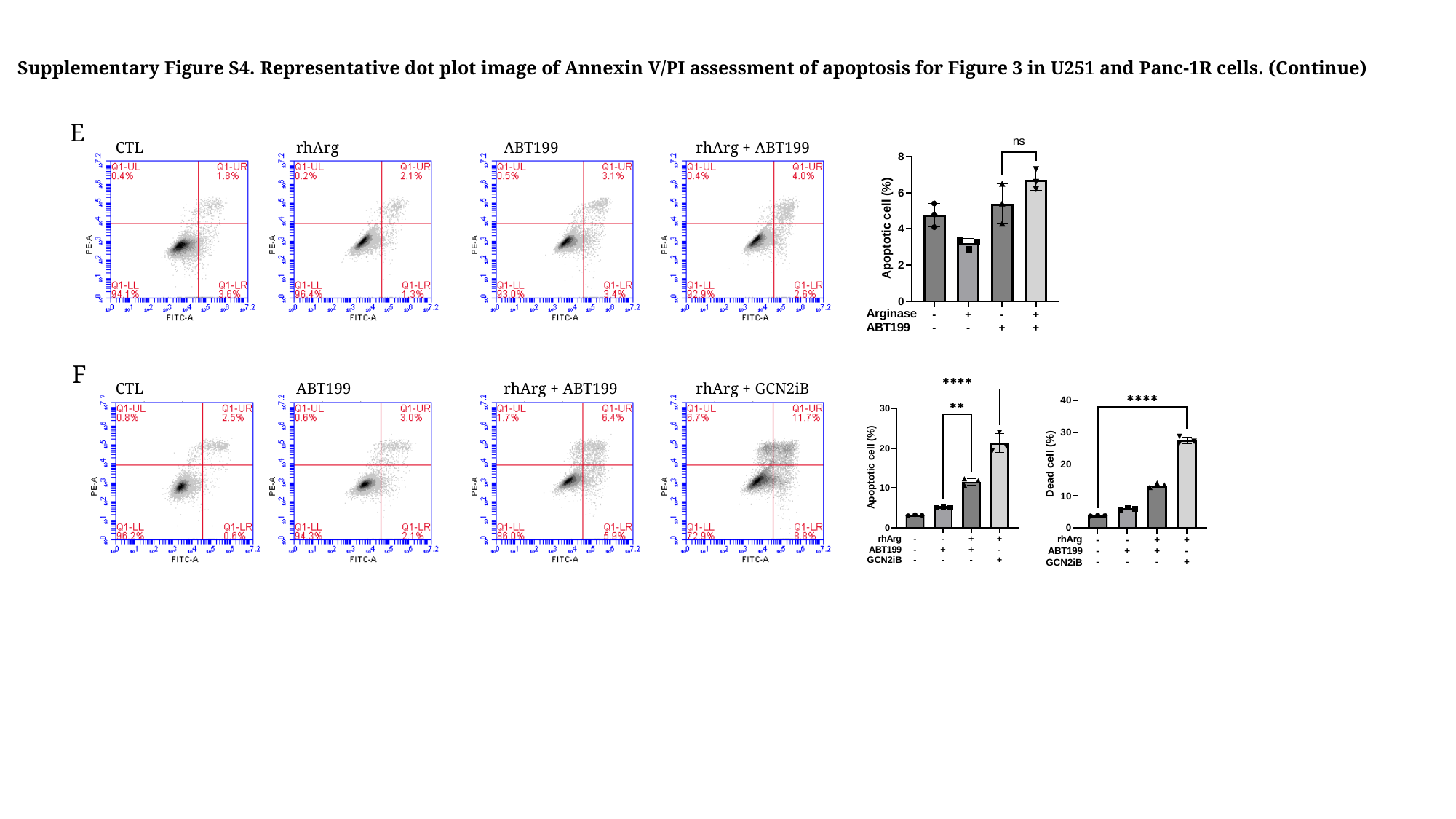

Supplementary Figure S4. Representative dot plot image of Annexin V/PI assessment of apoptosis for Figure 3 in U251 and Panc-1R cells. (Continue)
E
CTL
rhArg
ABT199
rhArg + ABT199
F
CTL
ABT199
rhArg + ABT199
rhArg + GCN2iB

## Slide 9
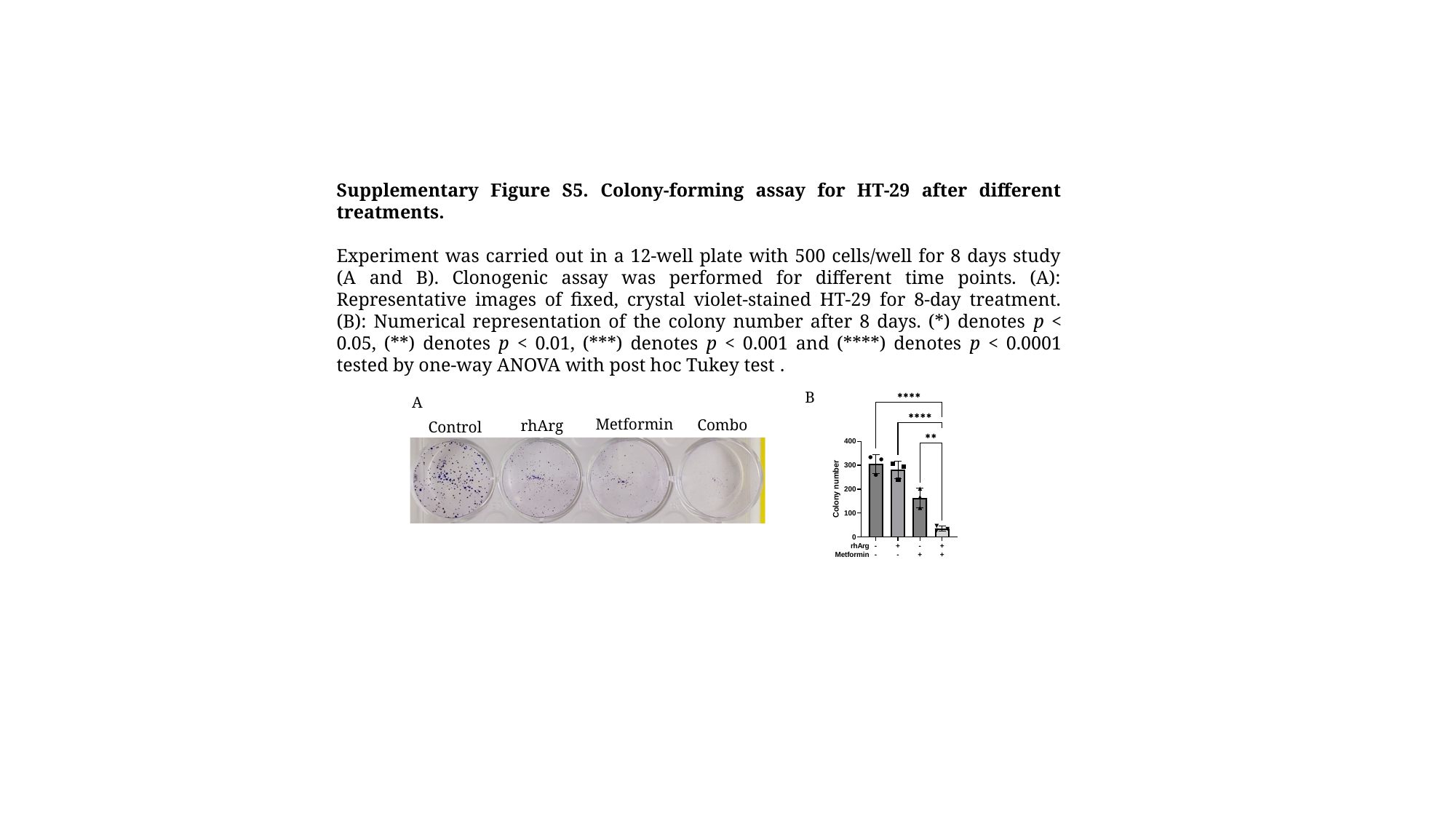

Supplementary Figure S5. Colony-forming assay for HT-29 after different treatments.
Experiment was carried out in a 12-well plate with 500 cells/well for 8 days study (A and B). Clonogenic assay was performed for different time points. (A): Representative images of fixed, crystal violet-stained HT-29 for 8-day treatment. (B): Numerical representation of the colony number after 8 days. (*) denotes p < 0.05, (**) denotes p < 0.01, (***) denotes p < 0.001 and (****) denotes p < 0.0001 tested by one-way ANOVA with post hoc Tukey test .
B
A
Metformin
Combo
rhArg
Control

## Slide 10
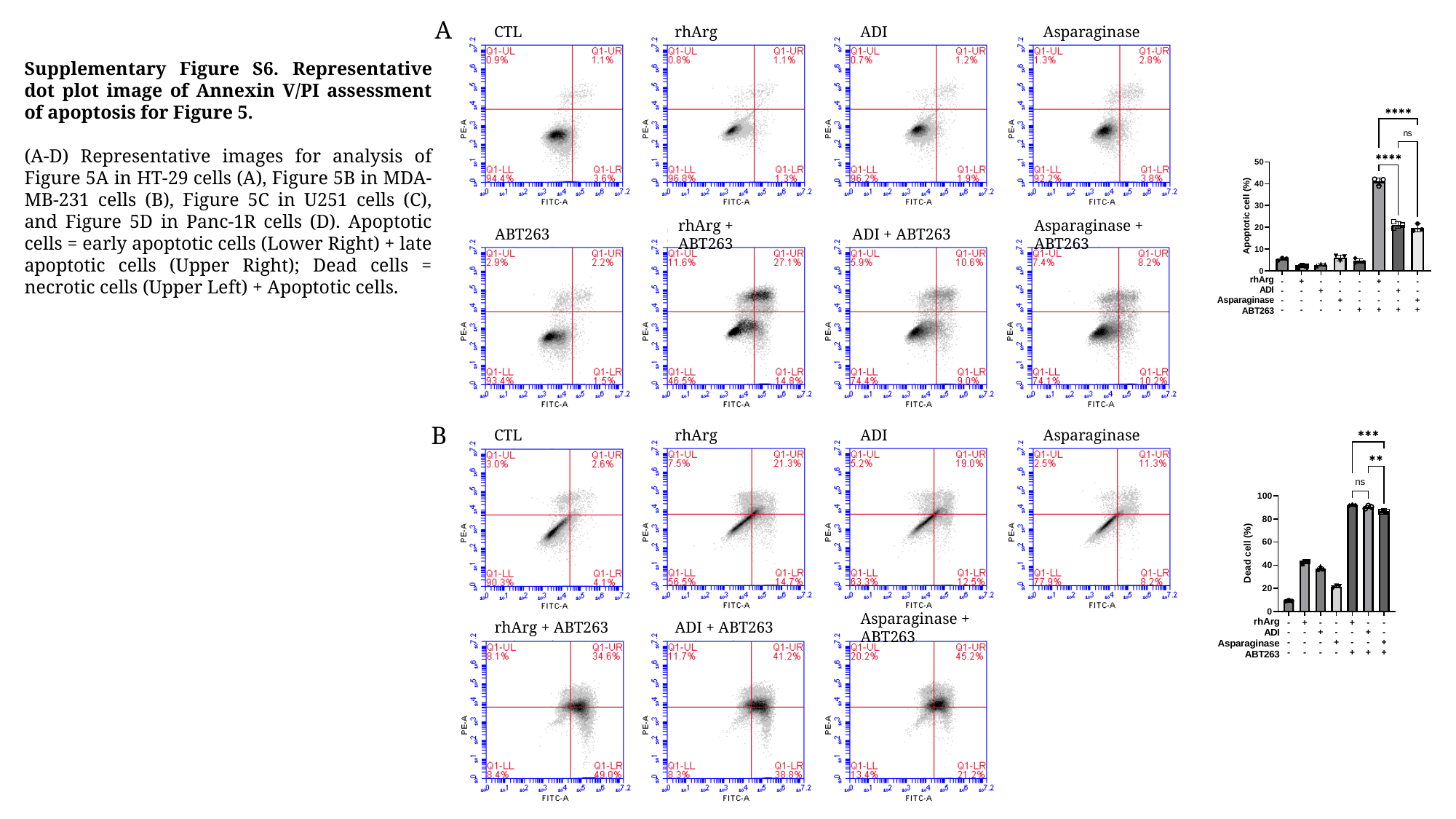

A
CTL
rhArg
ADI
Asparaginase
Supplementary Figure S6. Representative dot plot image of Annexin V/PI assessment of apoptosis for Figure 5.
(A-D) Representative images for analysis of Figure 5A in HT-29 cells (A), Figure 5B in MDA-MB-231 cells (B), Figure 5C in U251 cells (C), and Figure 5D in Panc-1R cells (D). Apoptotic cells = early apoptotic cells (Lower Right) + late apoptotic cells (Upper Right); Dead cells = necrotic cells (Upper Left) + Apoptotic cells.
ABT263
rhArg + ABT263
ADI + ABT263
Asparaginase + ABT263
B
CTL
rhArg
ADI
Asparaginase
rhArg + ABT263
ADI + ABT263
Asparaginase + ABT263

## Slide 11
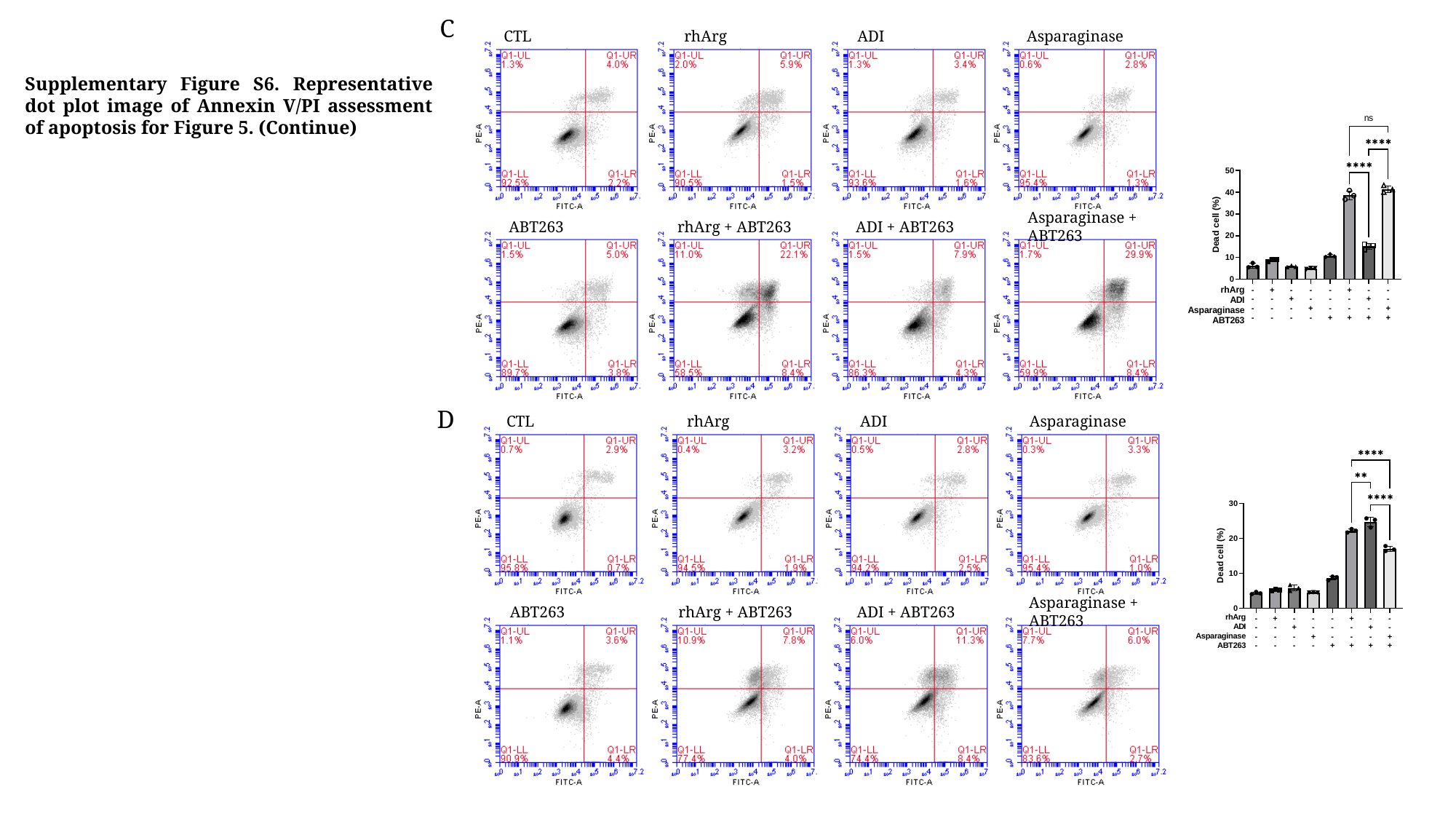

C
CTL
rhArg
ADI
Asparaginase
Supplementary Figure S6. Representative dot plot image of Annexin V/PI assessment of apoptosis for Figure 5. (Continue)
ABT263
rhArg + ABT263
ADI + ABT263
Asparaginase + ABT263
D
CTL
rhArg
ADI
Asparaginase
ABT263
rhArg + ABT263
ADI + ABT263
Asparaginase + ABT263

## Slide 12
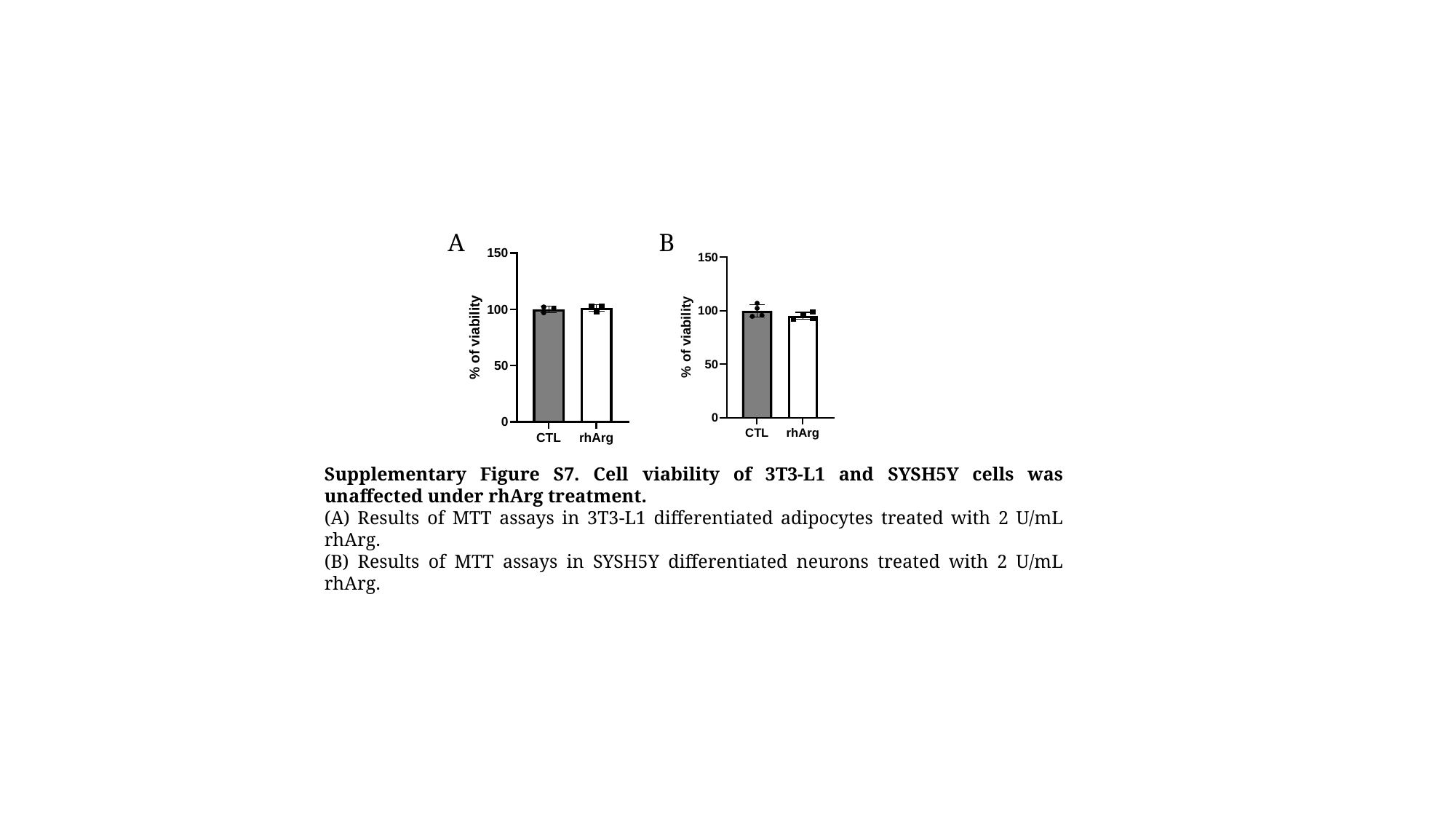

A
B
Supplementary Figure S7. Cell viability of 3T3-L1 and SYSH5Y cells was unaffected under rhArg treatment.
(A) Results of MTT assays in 3T3-L1 differentiated adipocytes treated with 2 U/mL rhArg.
(B) Results of MTT assays in SYSH5Y differentiated neurons treated with 2 U/mL rhArg.
