# Supplementary material for: The Synergistic Effects of rhArg with Bcl-2 Inhibitors or Metformin Co-Treatment in Multiple Cancer Cell Models
Source: Cells. 2026 Jan 16;15(2):164. doi: 10.3390/cells15020164 (PMC12839320; doi:10.3390/cells15020164)
Supplement: Supplementary file 1 [file cells-15-00164-s001.zip › File S1. Raw WB image (Final).pptx]

## Slide 1
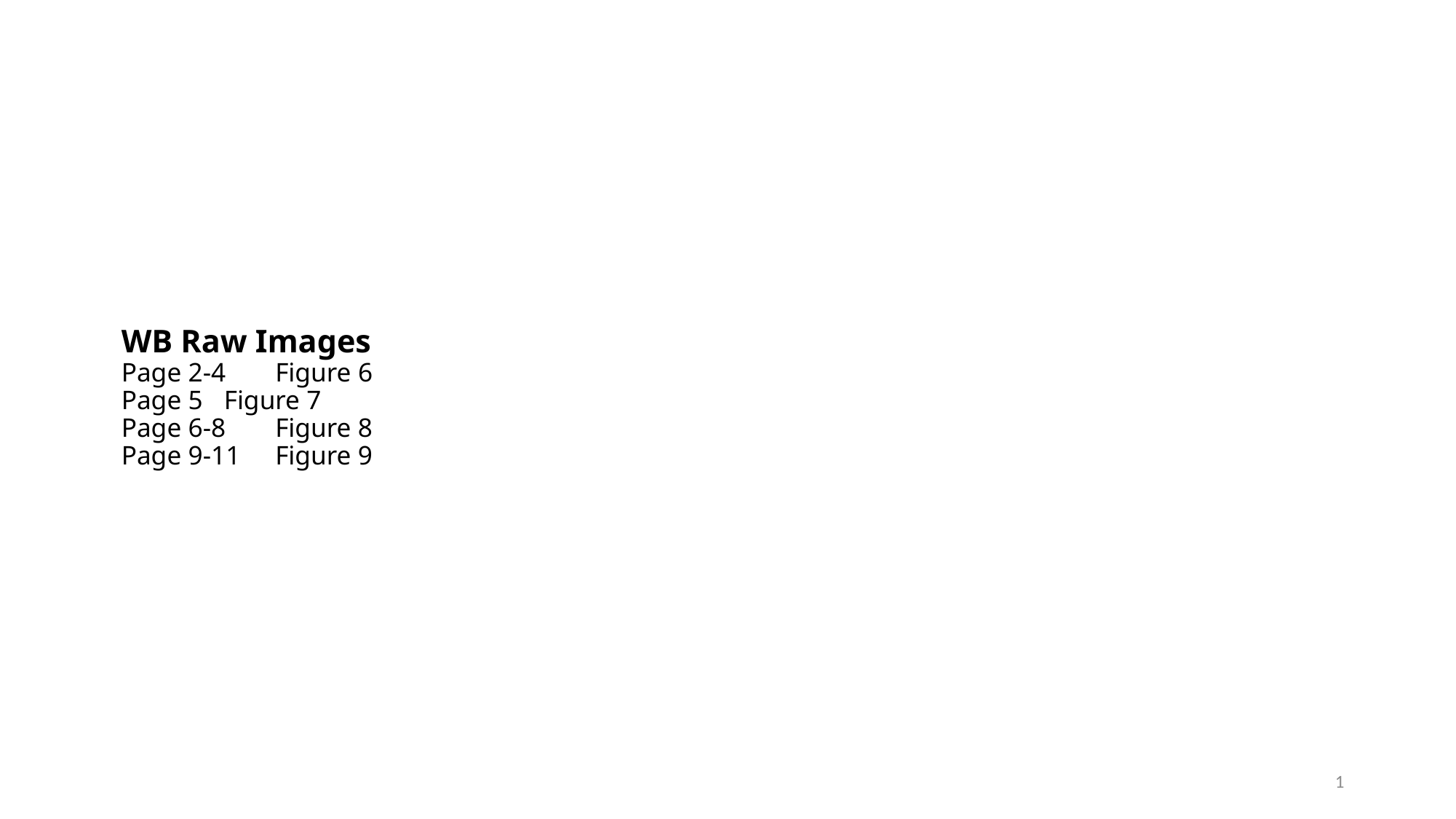

# WB Raw Images Page 2-4 		Figure 6 Page 5 		Figure 7 Page 6-8 		Figure 8 Page 9-11 		Figure 9
1

## Slide 2
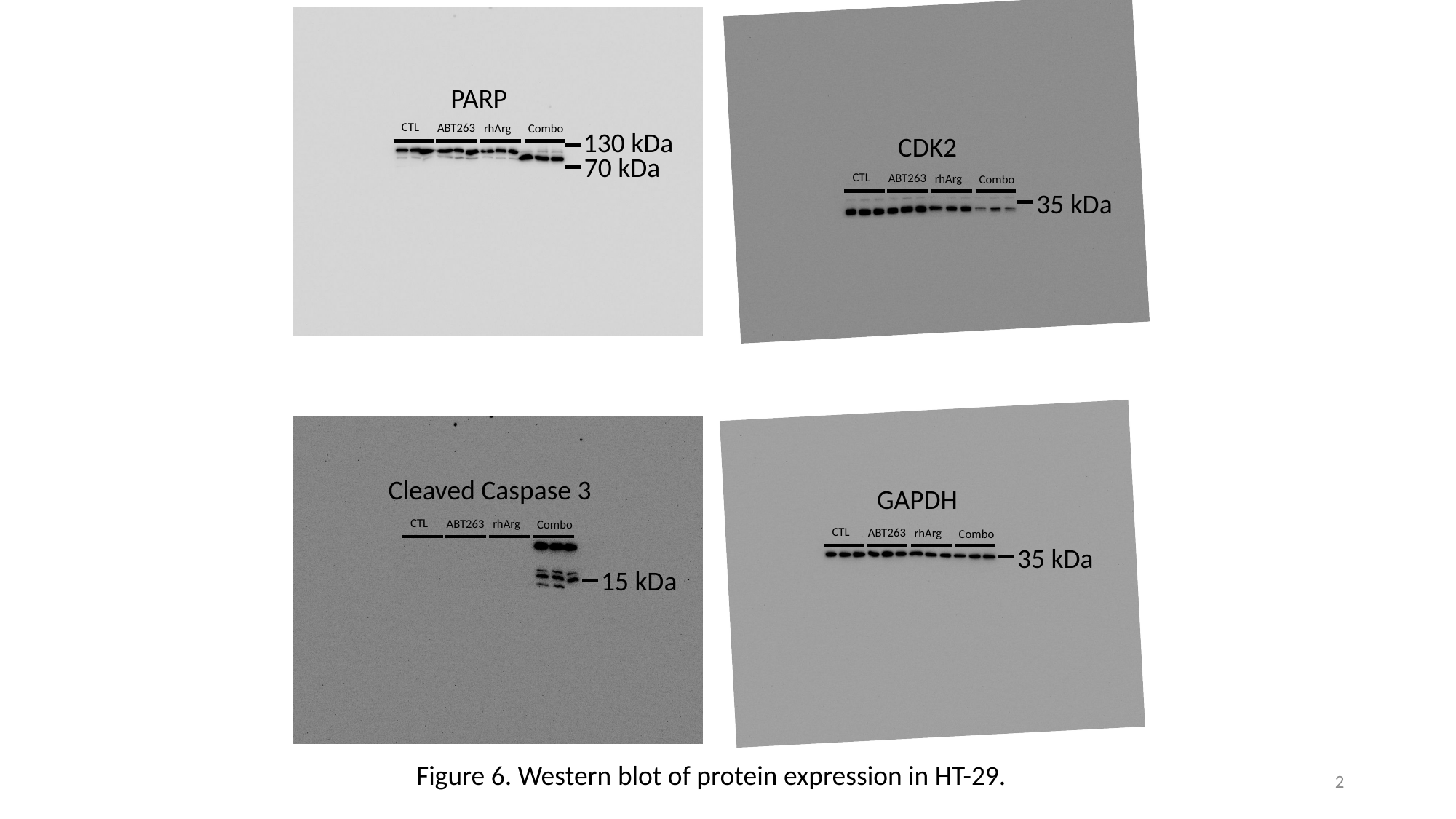

PARP
CTL
ABT263
rhArg
Combo
130 kDa
CDK2
70 kDa
CTL
ABT263
rhArg
Combo
35 kDa
Cleaved Caspase 3
GAPDH
CTL
ABT263
rhArg
Combo
CTL
ABT263
rhArg
Combo
35 kDa
15 kDa
Figure 6. Western blot of protein expression in HT-29.
2

## Slide 3
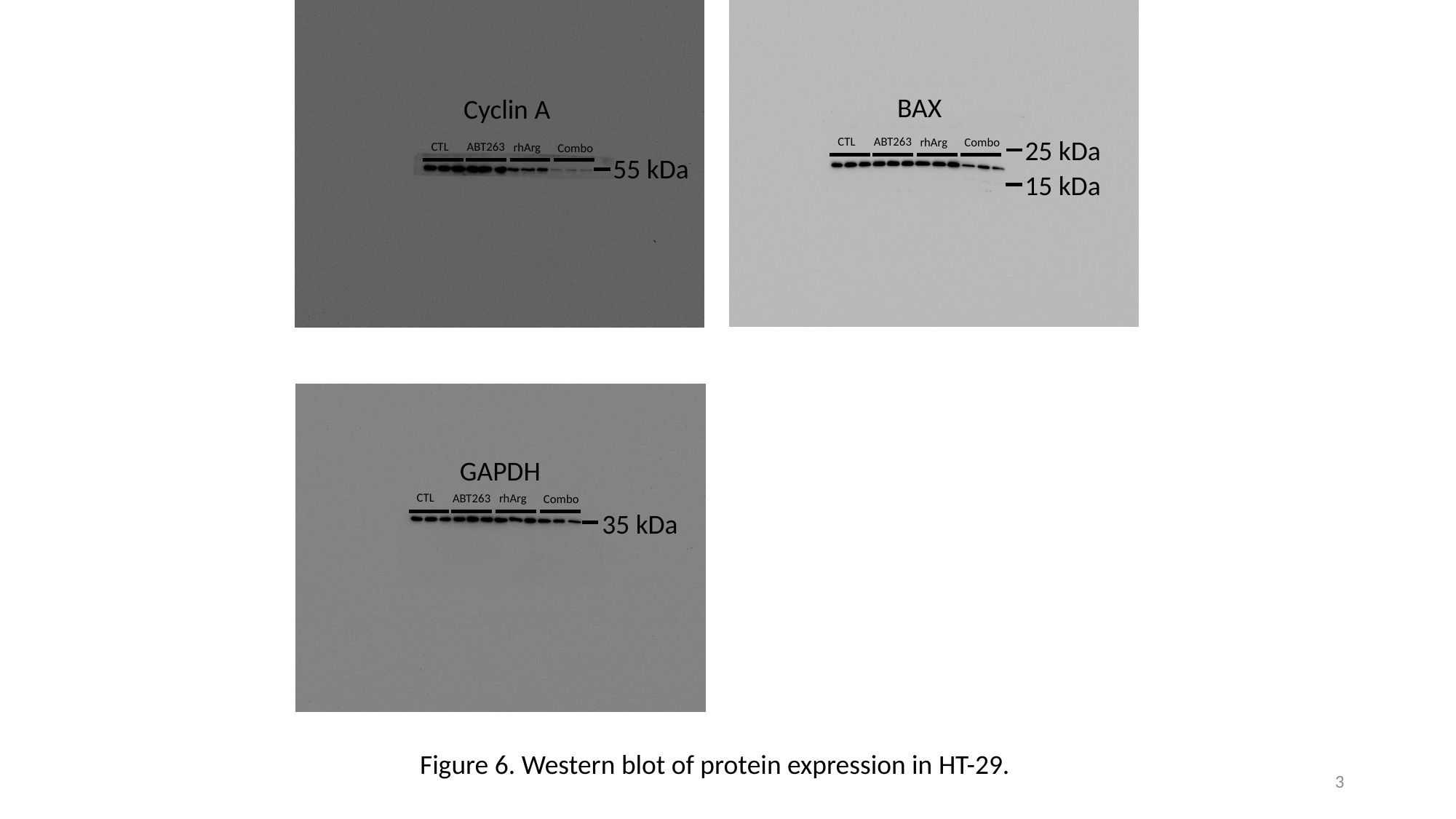

BAX
Cyclin A
25 kDa
CTL
ABT263
rhArg
Combo
CTL
ABT263
rhArg
Combo
55 kDa
15 kDa
GAPDH
CTL
ABT263
rhArg
Combo
35 kDa
Figure 6. Western blot of protein expression in HT-29.
3

## Slide 4
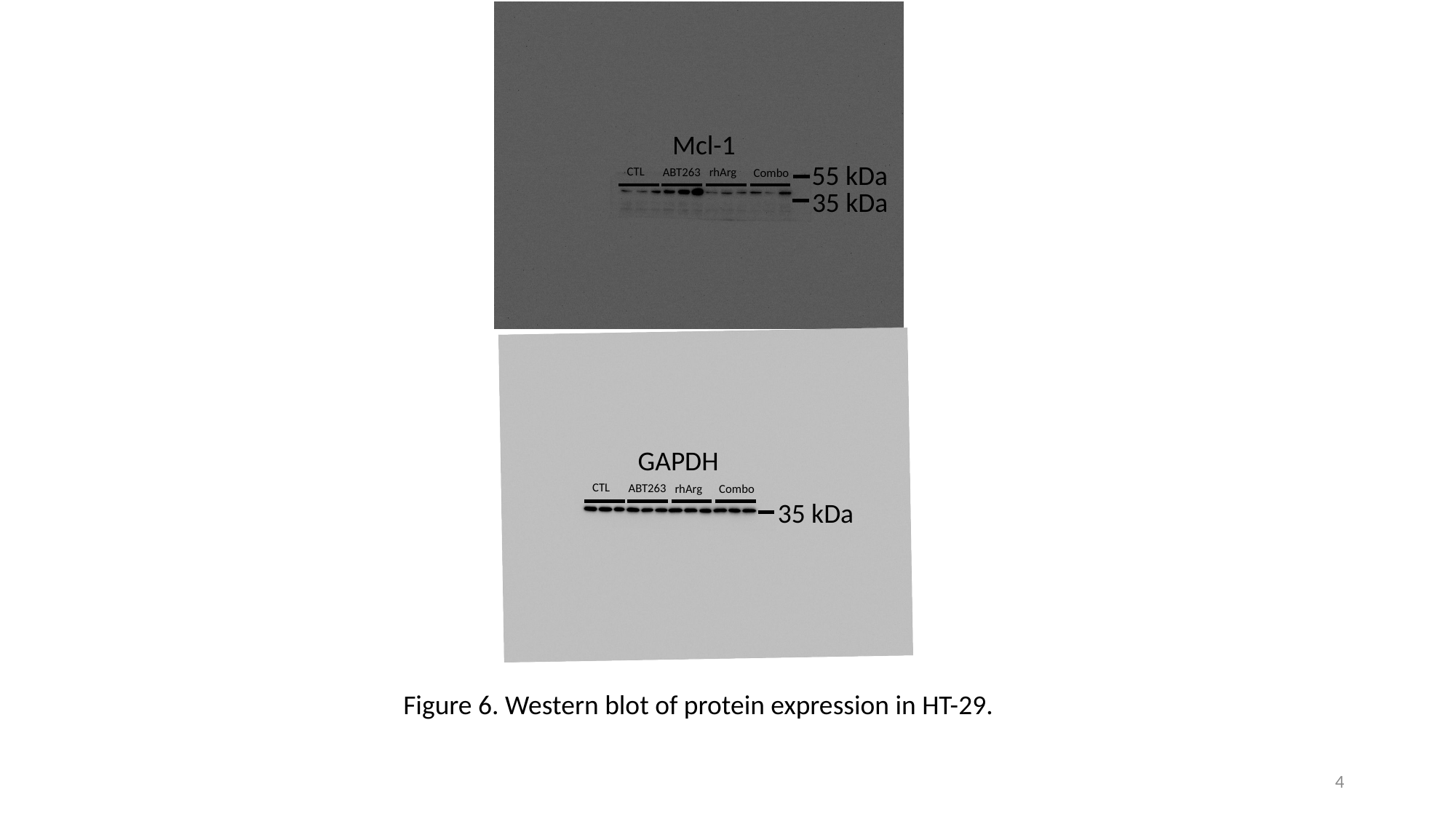

Mcl-1
55 kDa
CTL
ABT263
rhArg
Combo
35 kDa
GAPDH
CTL
ABT263
rhArg
Combo
35 kDa
Figure 6. Western blot of protein expression in HT-29.
4

## Slide 5
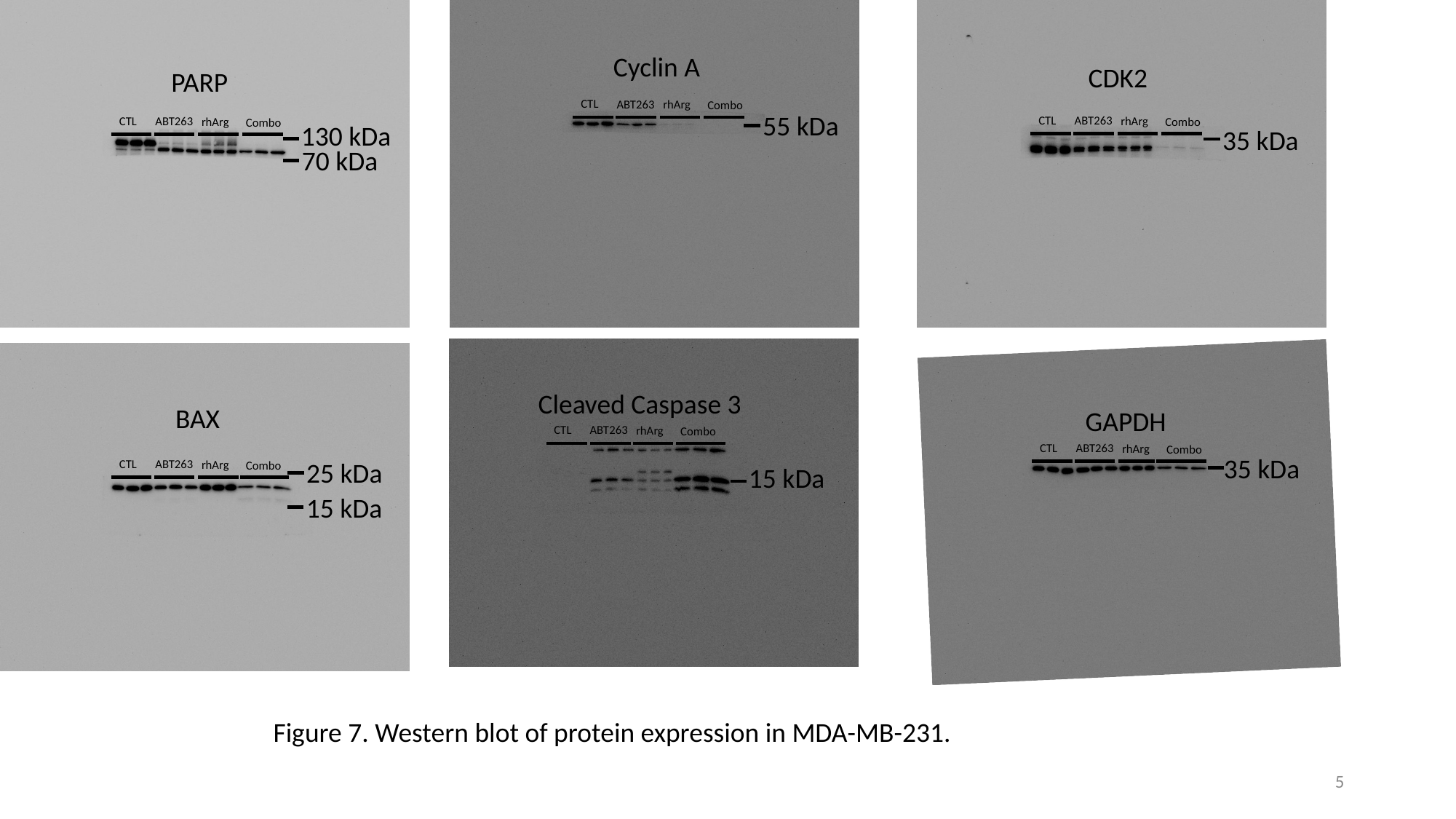

Cyclin A
CDK2
PARP
CTL
ABT263
rhArg
Combo
55 kDa
CTL
ABT263
CTL
rhArg
ABT263
Combo
rhArg
Combo
130 kDa
35 kDa
70 kDa
Cleaved Caspase 3
BAX
GAPDH
CTL
ABT263
rhArg
Combo
CTL
ABT263
rhArg
Combo
35 kDa
25 kDa
CTL
ABT263
rhArg
Combo
15 kDa
15 kDa
Figure 7. Western blot of protein expression in MDA-MB-231.
5

## Slide 6
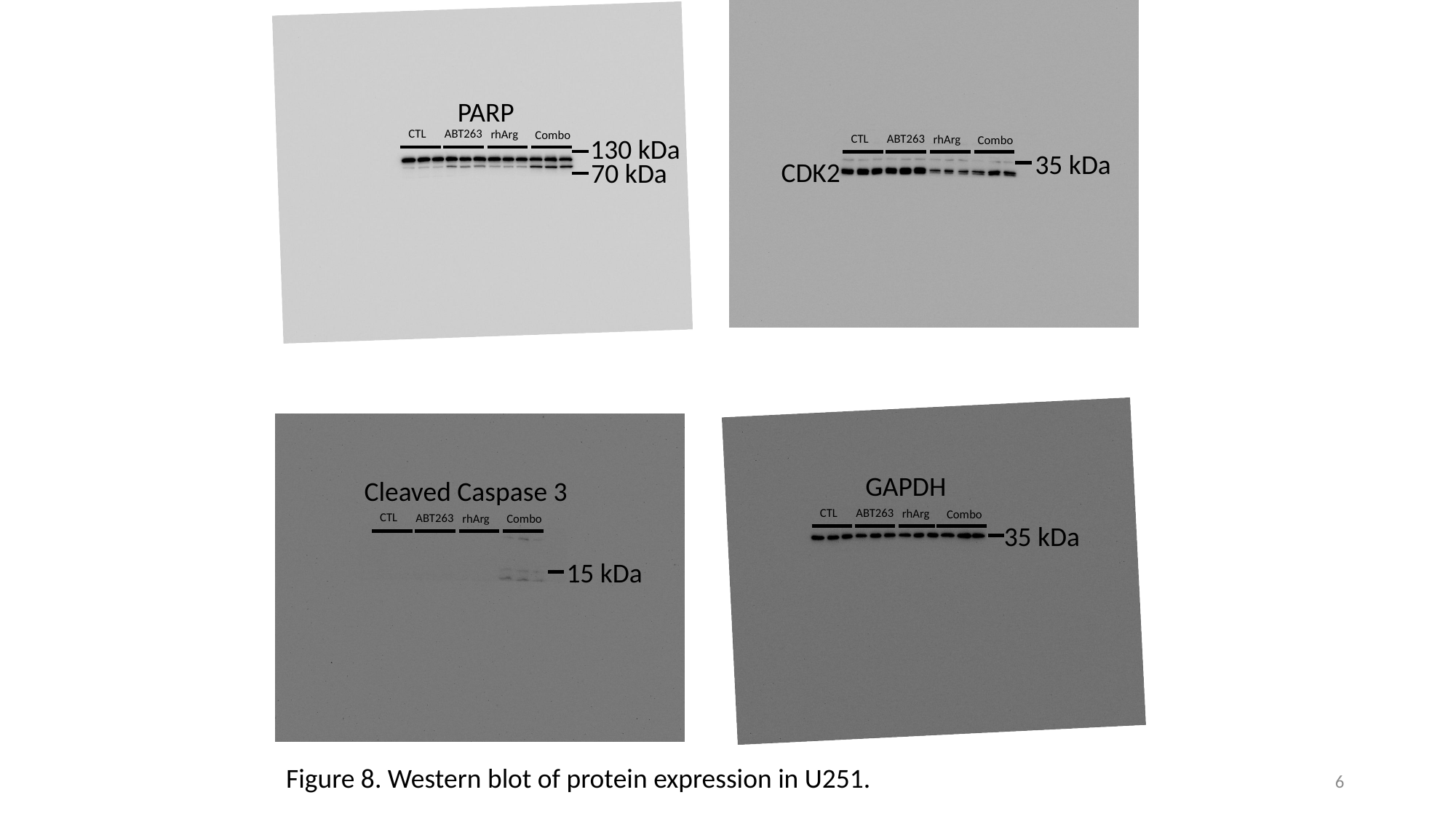

PARP
CTL
ABT263
rhArg
Combo
CTL
ABT263
rhArg
130 kDa
Combo
35 kDa
CDK2
70 kDa
GAPDH
Cleaved Caspase 3
CTL
ABT263
rhArg
Combo
CTL
ABT263
rhArg
Combo
35 kDa
15 kDa
Figure 8. Western blot of protein expression in U251.
6

## Slide 7
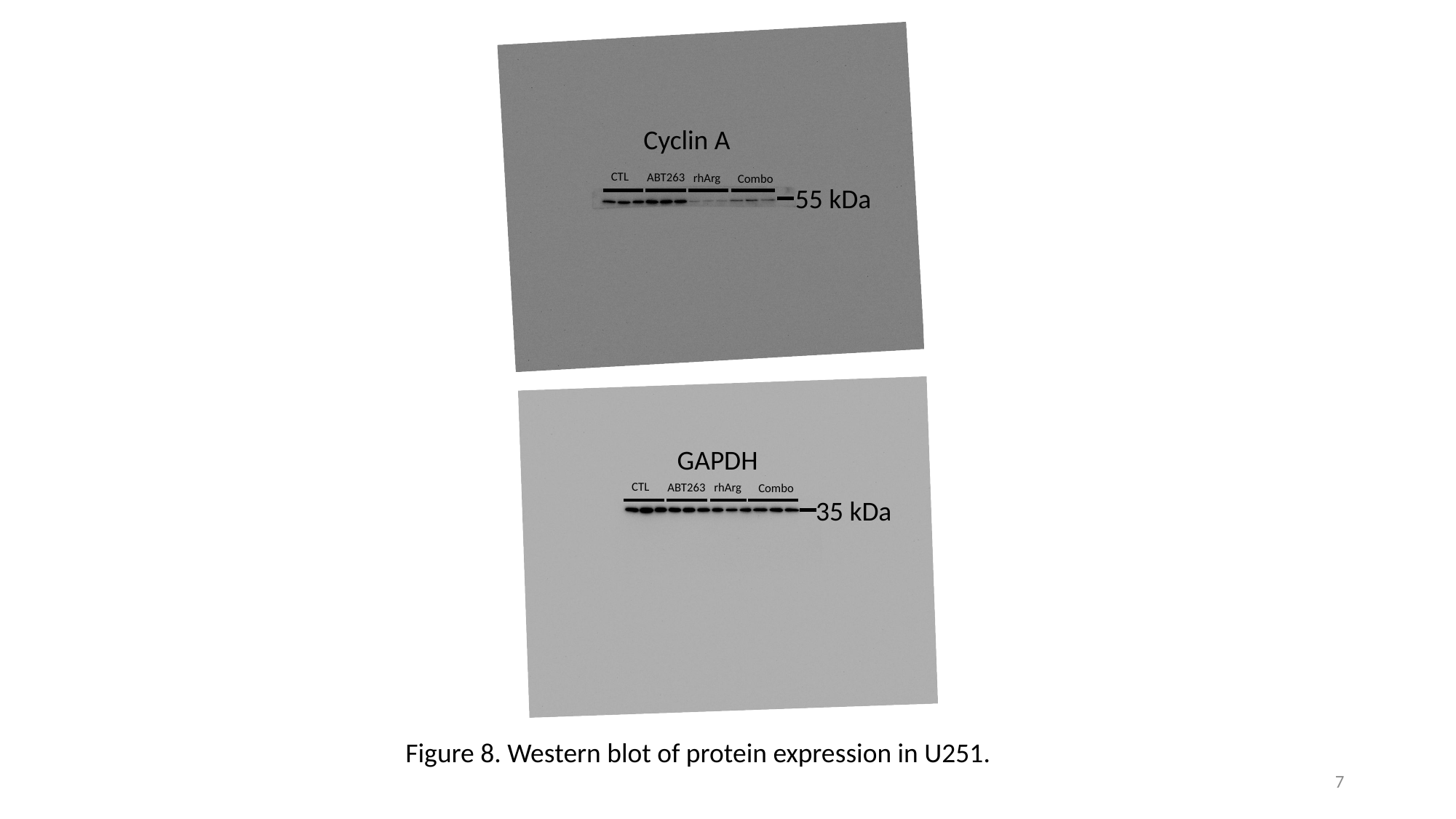

Cyclin A
CTL
ABT263
rhArg
Combo
55 kDa
GAPDH
CTL
ABT263
rhArg
Combo
35 kDa
Figure 8. Western blot of protein expression in U251.
7

## Slide 8
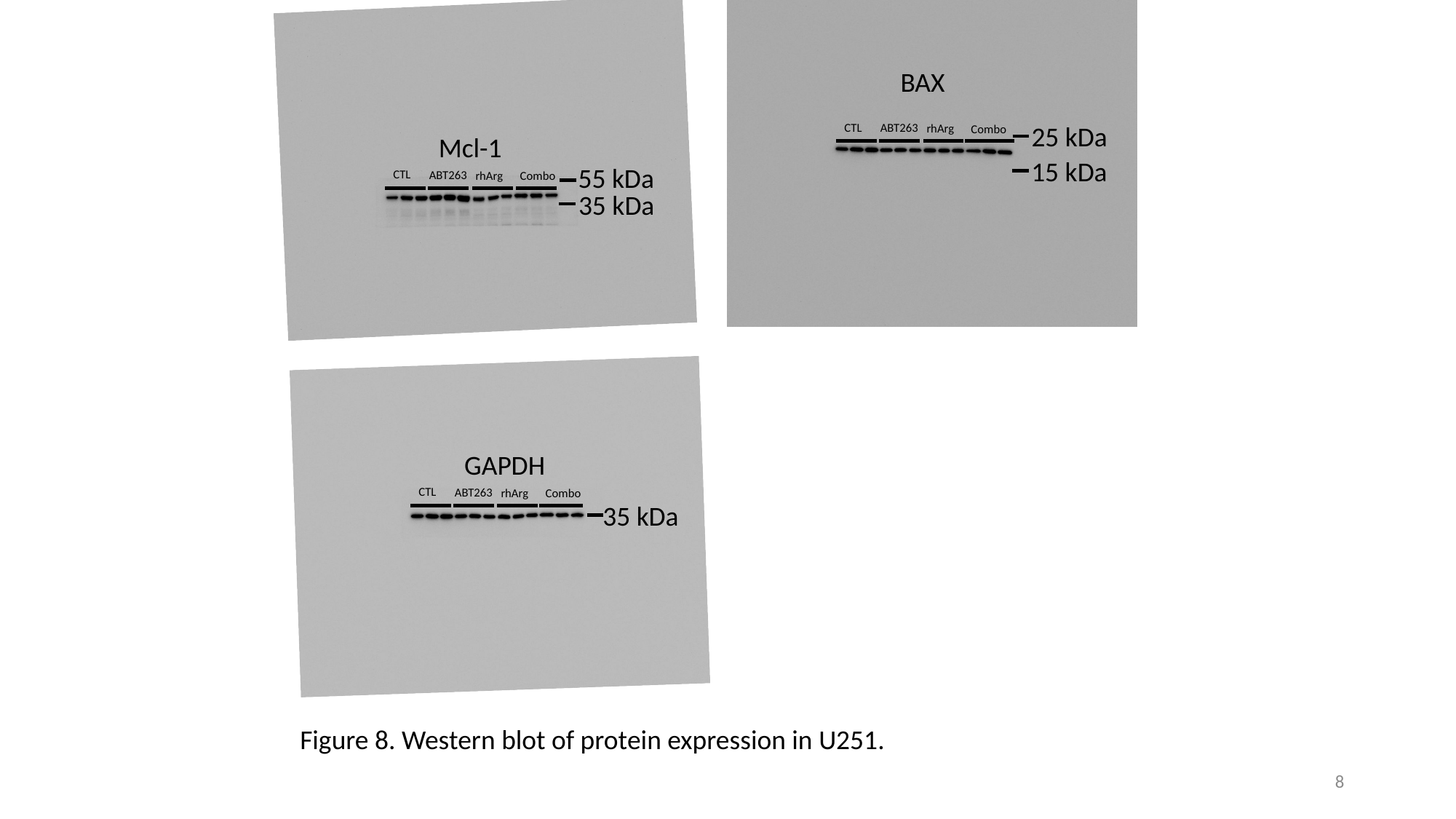

BAX
25 kDa
CTL
ABT263
rhArg
Combo
Mcl-1
15 kDa
55 kDa
CTL
ABT263
rhArg
Combo
35 kDa
GAPDH
CTL
ABT263
rhArg
Combo
35 kDa
Figure 8. Western blot of protein expression in U251.
8

## Slide 9
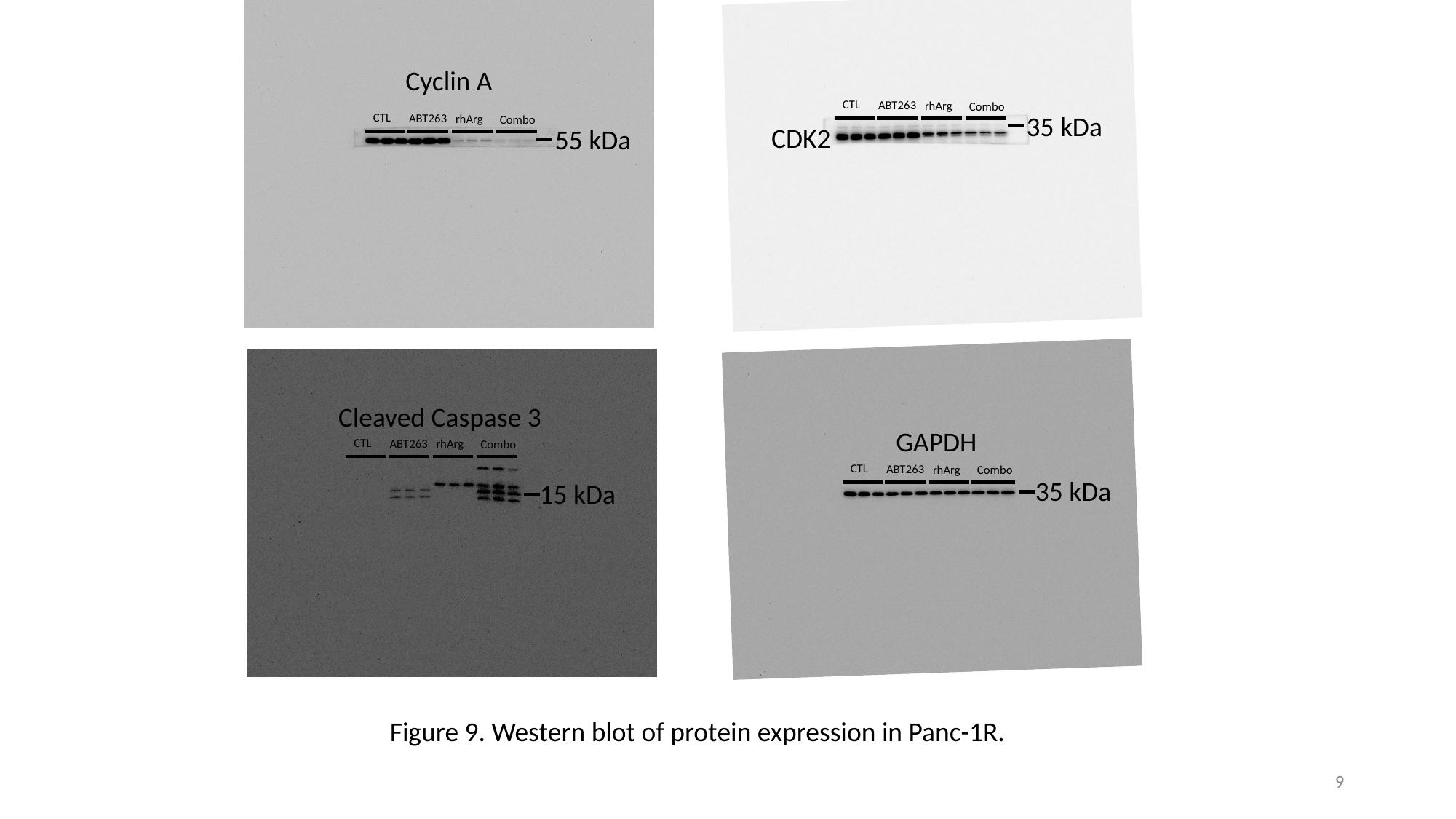

Cyclin A
CTL
ABT263
rhArg
Combo
35 kDa
CTL
ABT263
rhArg
Combo
CDK2
55 kDa
Cleaved Caspase 3
GAPDH
CTL
ABT263
rhArg
Combo
CTL
ABT263
rhArg
Combo
35 kDa
15 kDa
Figure 9. Western blot of protein expression in Panc-1R.
9

## Slide 10
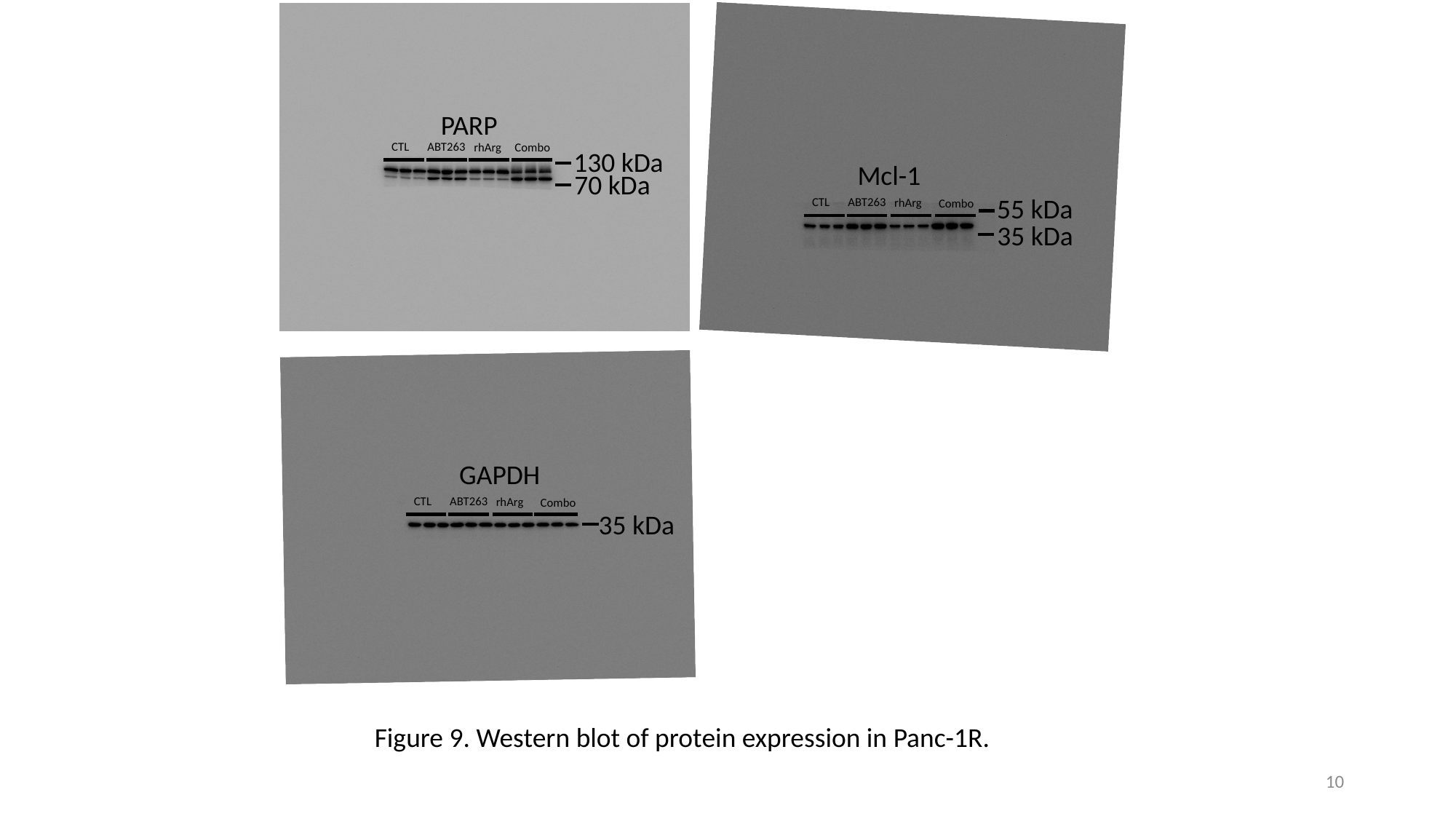

PARP
CTL
ABT263
rhArg
Combo
130 kDa
Mcl-1
70 kDa
55 kDa
CTL
ABT263
rhArg
Combo
35 kDa
GAPDH
CTL
ABT263
rhArg
Combo
35 kDa
Figure 9. Western blot of protein expression in Panc-1R.
10

## Slide 11
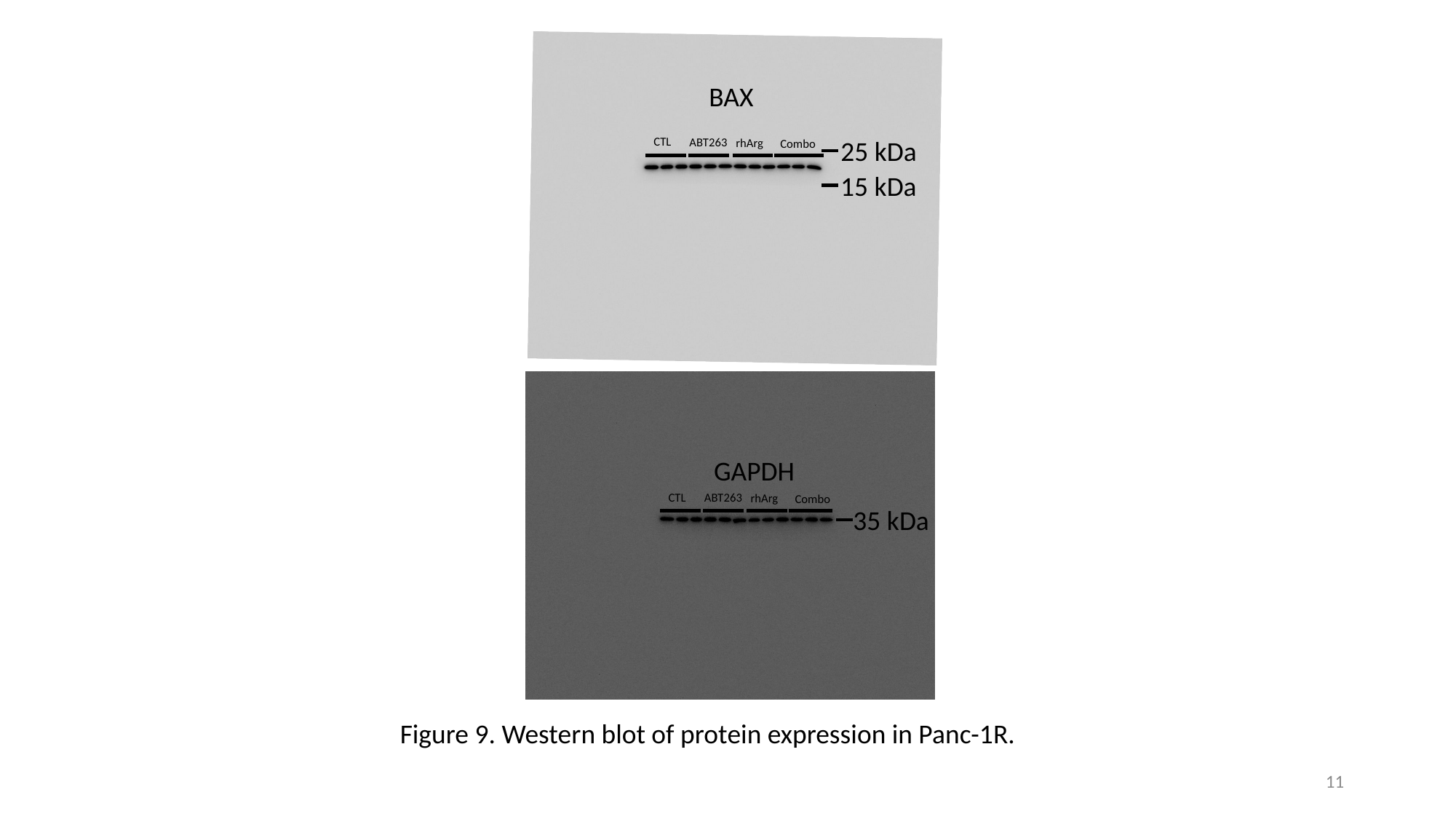

BAX
25 kDa
CTL
ABT263
rhArg
Combo
15 kDa
GAPDH
CTL
ABT263
rhArg
Combo
35 kDa
Figure 9. Western blot of protein expression in Panc-1R.
11
